# Large scale comparison of QSAR and conformal prediction methods and their applications in drug discovery

**DOI:** 10.1186/s13321-018-0325-4

**Published:** 2019-01-10

**Authors:** Nicolas Bosc, Francis Atkinson, Eloy Felix, Anna Gaulton, Anne Hersey, Andrew R. Leach

**Affiliations:** Chemogenomics Team, European Bioinformatics Institute (EMBL-EBI), Wellcome Genome Campus, Hinxton, Cambridge CB10 1SD UK

**Keywords:** QSAR, Mondrian conformal prediction, ChEMBL, Classification models, Cheminformatics

## Abstract

Structure–activity relationship modelling is frequently used in the early stage of drug discovery to assess the activity of a compound on one or several targets, and can also be used to assess the interaction of compounds with liability targets. QSAR models have been used for these and related applications over many years, with good success. Conformal prediction is a relatively new QSAR approach that provides information on the certainty of a prediction, and so helps in decision-making. However, it is not always clear how best to make use of this additional information. In this article, we describe a case study that directly compares conformal prediction with traditional QSAR methods for large-scale predictions of target-ligand binding. The ChEMBL database was used to extract a data set comprising data from 550 human protein targets with different bioactivity profiles. For each target, a QSAR model and a conformal predictor were trained and their results compared. The models were then evaluated on new data published since the original models were built to simulate a “real world” application. The comparative study highlights the similarities between the two techniques but also some differences that it is important to bear in mind when the methods are used in practical drug discovery applications.

**Electronic supplementary material:**

The online version of this article (10.1186/s13321-018-0325-4) contains supplementary material, which is available to authorized users.

## Introduction

Public databases of bioactivity data play a critical role in modern translational science. They provide a central place to access the ever-increasing amounts of data that would otherwise have to be extracted from tens of thousands of different journal articles. They make the data easier to use by automated and/or manual classification, annotation and standardisation approaches. Finally, by making their content freely accessible, the entire scientific community can query, extract and download information of interest. As a result, such public resources have been instrumental in the evolution of disciplines such as data mining and machine learning [1]. PubChem and ChEMBL represent the two largest public domain databases of molecular activity data [2]. The latest release (version 24) of ChEMBL (ChEMBL_24) contains more than 6 million curated data points for around 7500 protein targets and 1.2 million distinct compounds [3]. This represents a gold mine for chemists, biologists, toxicologists and modellers alike.

Contemporary experimental approaches and publication norms mean that the ChEMBL database is inherently sparsely populated with regard to the compound/target matrix. Therefore, in silico models are particularly useful, as they can in principle be used to predict activities for protein-molecule pairs that are absent from the public experimental record and the compound/target data matrix. Quantitative structure–activity relationship (QSAR) models have been used for decades to predict the activities of compounds on a given protein [1, 4, 5]. These models are then frequently used for selecting compound subsets for screening and to identify compounds for synthesis, but also have other applications ranging from prediction of blood–brain barrier permeation [6] to toxicity prediction [7]. These many applications of QSAR not only differ in their scope but also in terms of the level of confidence required for the results to be practically useful. For example, it could be considered that compound selection for screening may tolerate a lower level of confidence than synthesis suggestions due to the inherently higher cost of the latter.

Traditional QSAR and machine learning methods suffer from the lack of a formal confidence score associated with each prediction. The concept of a model’s applicability domain (AD) aims to address this by representing the chemical space outside which the predictions cannot be considered reliable [8–10]. However, the concept of chemical space can be fuzzy and it is not always straightforward to represent its boundaries. Recently, some new techniques have been introduced which aim to address this issue of confidence associated with machine learning results. In this article we focus on conformal prediction (CP) [11], but recognise that there are also alternatives such as Venn–ABERS predictors [12, 13] which have also been applied to drug discovery applications [14–16]. As with QSAR, these approaches rely on a training set of compounds characterised by a set of molecular descriptors that is used to build a model using a machine learning algorithm. However, their mathematical frameworks differ—QSAR predictions are the direct outputs of the model whereas CP and Venn–ABERS rely on past experience provided by a calibration set to assign a confidence level to each prediction.

The mathematical concepts behind CP have been published by Vovk et al. [11, 17] and the method has been described in the context of protein-compound interaction prediction by Norinder et al. [18]. Several examples of CP applications applied in drug discovery [18–21] or toxicity prediction have also been reported [22–25]. In practice, it is common to observe the results using different confidence levels and to decide, a posteriori, with what confidence a CP model can be trusted.

In this study, the development of QSAR and CP models for a large number of protein targets is described and the differences in their predictions is examined. We used the data available in the ChEMBL database for this purpose. As we will describe later in this paper, the general challenges with such an application are that sometimes there are limited number of data points available and there is an imbalance between the activity classes. This then requires a compromise to be achieved between the number of models that can be built, the numbers of data points used to build each model, and model performance. This is unfortunately a situation very common in drug discovery where predictive models can have the biggest impact early in a project when (by definition) there may be relatively few data available. As described later, in this study we used machine learning techniques able to cope with these limitations, specifically class weighting for QSAR and Mondrian conformal prediction (MCP) [26]. Finally, we aim to compare QSAR and MCP as objectively as possible, making full use of all the data, subject to the constraints inherent in each method.

## Methods

### Data sets

**Fig. 1:**
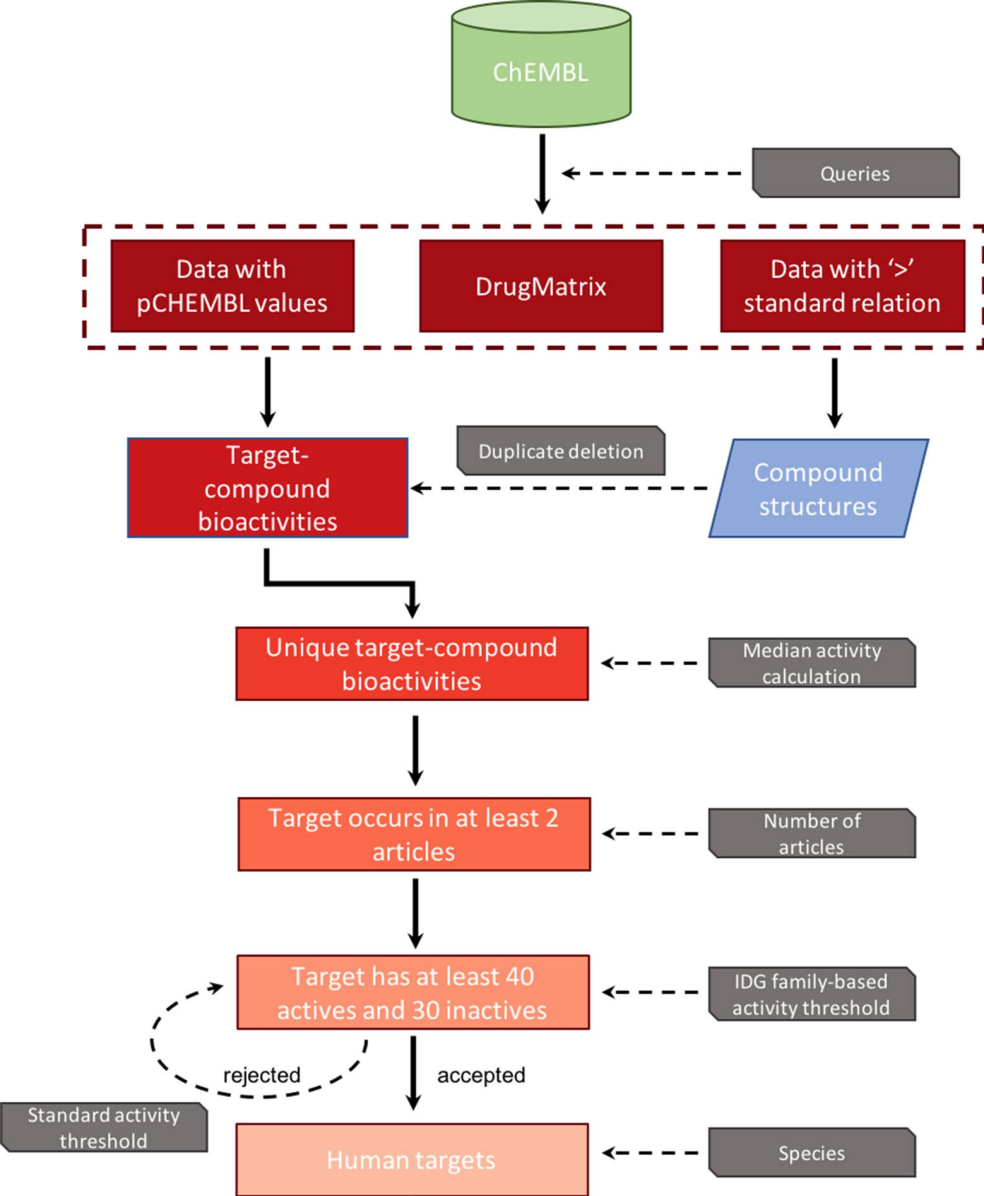
Schema of the data collection from ChEMBL

Data were extracted from version 23 of the ChEMBL database (ChEMBL_23) [27] using a protocol adapted from the study of Lenselink et al. [24] (Fig. 1). First, human targets flagged as ‘SINGLE PROTEIN’ or ‘PROTEIN COMPLEX’ with confidence scores of 9 and 7, respectively, were selected. These scores indicate a definitive link between the protein and the species. More detail about the protein target definitions in ChEMBL is available elsewhere [28]. For each target, only bioactivities with pChEMBL values were chosen. This term refers to all the comparable measures of half-maximal responses (molar IC50, XC50, EC50, AC50, Ki, Kd, potency and ED50) on a negative logarithmic scale [28]. It is calculated only when the standard relation is known to be ‘=’. In addition, a set of high quality inactive data was extracted to improve the balance between active and inactive data in the models. The inactive data were selected considering pChEMBL-like activities (i.e. of the same activity types aforementioned) and only differ from the pChEMBL values by their standard relation being ‘<’ (Fig. 1).

We further restricted the selection to data extracted from the scientific literature (src_id = 1 in the database). Only activities that were not flagged as potential duplicates, with no data_validity_comment and whose activity_comment is not ‘inconclusive’ or ‘undetermined’ were considered.

In addition, the DrugMatrix molecular pharmacology profiles were included in the training set (src_id = 15 in the ChEMBL database) (Fig. 1) [29]. Because this data set is homogeneous, no particular filtering was required except for the activity type. Both Ki and IC50 values are provided for each protein-compound pair in the DrugMatrix binding assay subset. After verification it appeared that both values are systematically close so we chose to use the IC50 values.

For further validation of the models, the most recent version of ChEMBL (ChEMBL_24) was used as a temporal validation set by extracting activities for compounds that were not present in previous releases. The targets were restricted to those for which models were built using CHEMBL_23 data.

All the data, except those from ChEMBL_24, were grouped together using protein-compound pair information, and treated as one data set. The data from ChEMBL_24 were processed separately but exactly the same protocol was applied.

### Data preparation

Compound structures were extracted from the database in SMILES format and using RDKit (version 2017_09_01) [30], non stereospecific SMILES were calculated for each molecule. This means that stereoisomers have the same SMILES. We recognise that stereochemistry is a fundamental aspect of molecular activity and there are many examples of drugs with inactive enantiomers (e.g. dextro- and levo-cetirizine are inactive and active, respectively [31]). However, the 2D descriptors that we are using (see below) cannot differentiate these cases and, in the end, this approximation affects only about 1% of the total number of target-compound pairs extracted for this study.

When identical target-compound pairs were found, either because several measurements are found in the database or because of the stereochemical simplification described above, the median activity value was calculated. This step prevents duplicating the number of distinct structures available for each model and the risk of having the same compound in the training and the test set.

In order to promote structural diversity, targets were only retained if they were found in at least two different publications. Activities were assigned to active and inactive classes according to their protein family using activity thresholds as defined by the Illuminating the Druggable Genome consortium (IDG) [32] (Table 1). We treated each target as follows:

1. If the target had at least 40 active and 30 inactive compounds using the criteria in Table 1, it was retained for modelling;
2. If the protein target did not match condition (1) the compounds were divided into active/inactive sets using a default activity threshold of 6.5 logarithmic value units. If this enabled the target to meet criterion (1) then the protein target was retained. This threshold was shown to provide a balanced distribution of active and inactive compounds in the version 20 of ChEMBL [33], and this trend was confirmed for ChEMBL_23 (data not shown);
3. If the protein target did not match any of the previous criteria then it was discarded.

**Table 1:** Illuminating the Druggable Genome protein family activity thresholds

Protein families	Activity thresholds in logarithmic values (≥)
Protein kinases	7.5
G protein-coupled receptors	7
Nuclear receptors	7
Ion channels	5
Non-IDG protein families	6

We note that a number of approximations have been introduced in the approach described in this section. This reflects the focus of this study which is to build several hundreds of models involving (tens of) thousands of data points. This does differ from detailed model building involving just a single individual target, where a more bespoke approach to data preparation might be applied.

### Molecular descriptors

Molecular descriptors were calculated using RDKit. Morgan fingerprints were calculated with a radius of 2 and a length of 2048 bits [34]. In addition, six physicochemical descriptors were calculated using the Descriptors module: molecular weight (MolWt), number of hydrogen bond donors (NumHDonors), number of hydrogen bond acceptors (NumHAcceptors), number of rotatable bonds (NumRotatableBonds), lipophilicity (MolLogP) and the topological polar surface area (TPSA). These six physicochemical descriptors were scaled between 0 and 1 using the MinMaxScaler function provided by Scikit-learn version 0.19 [35].

### Model building

We chose to build simple active/inactive classification models. Although both QSAR and MCP can generate regression models, the numerous sources that populate the ChEMBL database result in data heterogeneity and potential uncertainties in quantitative activity values. When attempting prediction on multiple targets independently (as in this work), we consider the use of classification modelling to be a reasonable simplification of the problem.

QSAR and MCP classification models were built using the Random Forest (RF) method as implemented in Python by Scikit-learn version 0.19 [35] and the conformal prediction framework was developed using the nonconformist package version 2.1.0 [36]. The number of trees and the maximum depth of the tree, were set to values of 300 and 20 respectively. All other parameters were set to their default values. Internal tuning experiments using grid search demonstrated that these values generally enable us to obtain the most accurate models (data not shown).

For each target, two models were created: one QSAR model and one MCP. For QSAR, the RF models were trained using a training set that is then used to predict the class of each compound in the test set. The predictions are compared to the actual values to assess the predictivity of the model.

In CP, a machine learning model is trained and then applied to a calibration set containing active and inactive compounds. This returns a set of probabilities associated with each class (the *non*-*conformity scores*). When a new compound is predicted by the conformal predictor, the probability that it belongs to each class is calculated. These probabilities are compared to the lists of non-conformity scores to infer *p* values by calculating the number of non-conformity scores that are lower than the probability of the new compound, divided by the total number of compounds in the list. To be assigned to a specific class, the corresponding *p* value must be greater than a user-defined significance level (ε). Hence, new compounds are predicted as being in either one or the other class (single class prediction), in ‘both’ classes, or in none of them (‘empty’ class). Note that a CP result is often associated to a confidence level defined by 1 − ε and expressed as a percentage.

To deal with the imbalanced data sets in our panel, we considered parameters that aim to reduce the consequences of this on the predictions. In RF modelling, it is possible to assign different weights to each class to compensate for differences in the number of observations. We therefore set the RF parameter ‘class_weight’ to ‘balanced’. There is a variant of CP which can be utilised with imbalanced data called Mondrian conformal prediction (MCP) [19, 26]. This variant addresses the potential issue that can occur when a class is overrepresented and influences the prediction, resulting in the minority class being wrongly predicted. In this situation, the model might appear globally valid even if it is not the case for the underrepresented class. To deal with this issue, MCP divides data according to the classes and a separate significance level is applied for each of them. This helps to guarantee validity for each class.

### Model validation

To compare MCP to QSAR, for each target the data set was split into a training (80%) and a test set (20%) by applying a stratification sampling on the activity class. For MCP, the training set is further randomly divided into a proper training set (70%) and a calibration set (30%). For both techniques, exactly the same seed was applied when performing the first split so the test sets were the same for both techniques. The splitting procedure was repeated 100 times using the different random splits and the result for each compound was obtained by calculating the median probabilities for QSAR or *p* values for MCP, over the 100 predictions. For each iteration, particular attention was paid to perform exactly the same first split to enable comparisons to be made without introducing any bias due to the molecules present in the different sets. At this stage it appears that the training set of MCP is 30% smaller than for QSAR. Although this difference could favour QSAR, it was decided to apply this asymmetrical strategy to exploit 100% of the data available for each target as in a real-life modelling task.

For both QSAR and MCP, the internal performance was assessed for each model. The results were then grouped globally or by protein families to simplify the analysis. The sensitivity (ratio of the number of active compounds correctly classified to the total number of active compounds), specificity (ratio of the number of inactive compounds correctly classified to the total number of inactive compounds) and correct classification rate (CCR) which represents the mean of the two, were calculated for all the approaches.

While QSAR can return two single prediction classes, either ‘active’ or ‘inactive’, MCP can assign the compounds in two additional classes called ‘empty’ and ‘both’, depending on whether the conformal predictor cannot assign any class to the compound or whether it cannot discriminate between the classes. Whilst dual or no membership of the two activity classes may be considered unhelpful, this may still be useful for practical decision-making, depending on the degree of confidence required. Nevertheless, it may skew some of the comparisons we wish to make in this study. We therefore introduced three additional metrics (sensitivity_incl, specificity_incl and CCR_incl) when compounds assigned to the ‘both’ class are considered correctly classified, and three further metrics (sensitivity_excl, specificity_excl and CCR_excl) where compounds in the ‘both’ class are ignored.

In addition, for MCP the validity of the models was assessed. A MCP model is valid if the number of errors it commits does not exceed the chosen confidence level. The validity can also be calculated for each class individually to assess that they are both predicted with the same performance. In the context of validity measurement, compounds assigned either in the correct or in the ‘both’ classes are considered as correct.

External validation uses a subset of data that was left out of the model building. In this study, the prospective performance of all the models was addressed using a temporal validation approach as it is more representative of how models are used in practice [37]. Taking advantage of the features provided by the ChEMBL database, a temporal set was identified using version 24 of ChEMBL (ChEMBL_24) and predictions made using the QSAR and MCP models from ChEMBL_23 using the same protocols and metrics as for the internal validation.

## Results and discussion

### Modelling data set

Applying the selection protocol described in the Methods section above, a total of 550 human protein targets with varying numbers of data points were identified. The targets contain between 76 and 7707 unique compounds (and associated activities) with a mean of 742, a median of 391 and a first quartile of 184.

**Fig. 2:**
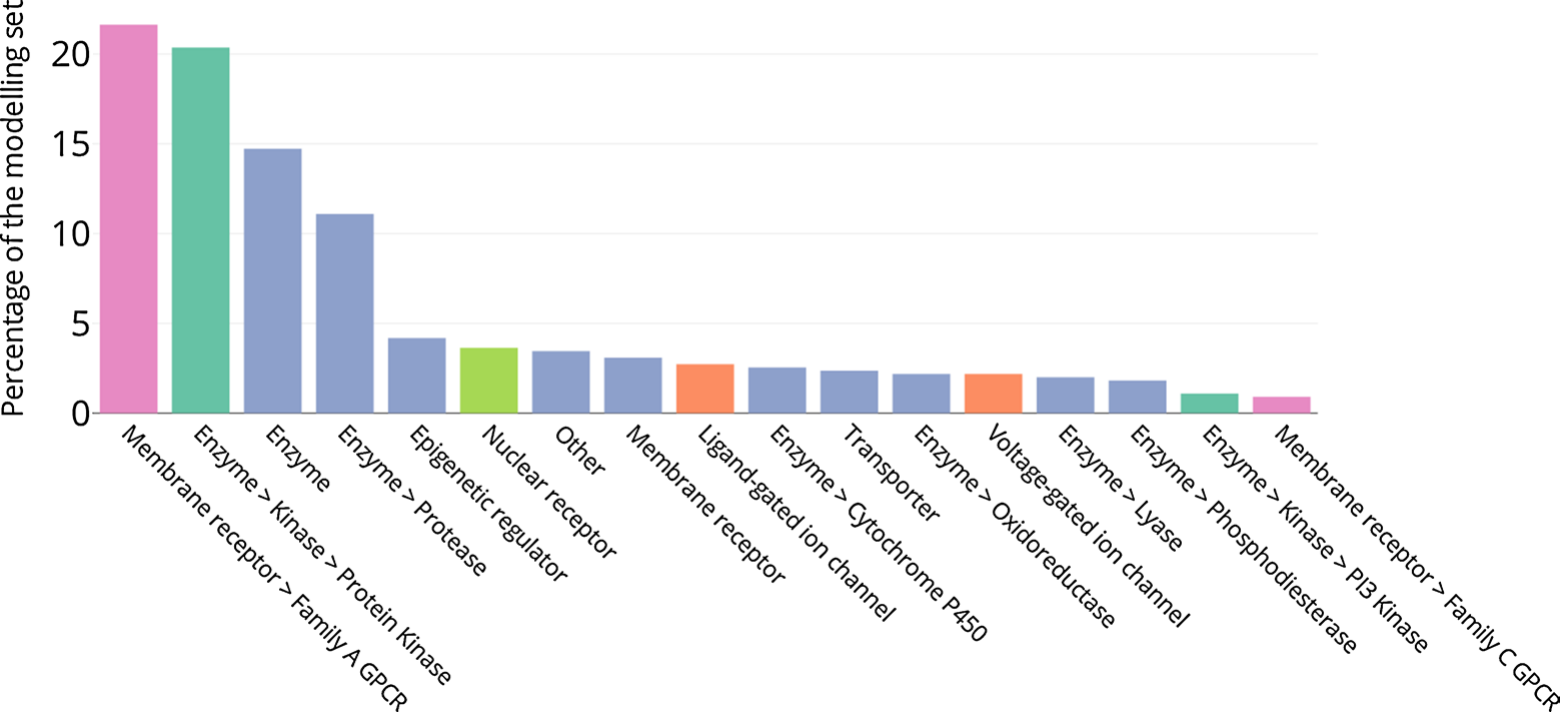
Percentage of the 550 selected targets by protein families. The protein family colours are the same for all the figures

Using the protein classification provided by the ChEMBL database, an analysis of the different protein families represented in this set was performed (Fig. 2). Family A G protein-coupled receptors (Rhodopsin-like) represent 21% of the selected targets, followed by the protein kinases (20%). Finding experimental data for these proteins is not surprising as they have been widely worked on for drug discovery and are the targets for many FDA-approved drugs [38–40]. 15% of the targets belong to the enzyme category which excludes protein kinase, protease, oxidoreductase, cytochrome P450, phosphodiesterase, lyase and phosphoinositol-3-kinase families that are considered separately. Other important families are proteases (11%), epigenetic regulators (4%) and nuclear receptors (3.6%). In total, these six protein families represent more than three quarters of the selected targets (Fig. 2). Details on the number of targets per protein families selected after each filtering step (see Methods) are presented in the Additional file 1: Table S1. It is also worth noting that 31 targets (6%) correspond to protein complexes and 78 (14%) targets have had their data selected not using the IDG activity thresholds. The full data sets used in this study is made available for download at ftp.ebi.ac.uk/pub/databases/chembl/qsar_vs_cp_modelling_data.

The ratio of active to inactive compounds for each target has a median value of 0.8 across all 550 targets with first and third quartile values of 0.39 and 1.59, respectively (Additional file 1: Figure S1). Hence, the data sets for the targets in our set are in general relatively well balanced but those at the margins may see their model performance struggling due to the class sizes, hence the strategies outlined above to cope with these situations for both QSAR and MCP are justified. Melanocorticoid receptor 5 (CHEMBL_ID: CHEMBL4608), interleukin-8 receptor A (CHEMBL_ID: CHEMBL4029) and melanocorticoid receptor 3 (CHEMBL_ID: CHEMBL4644) are the three proteins with the lowest ratio (< 0.05). At the opposite end, vanilloid receptor (CHEMBL_ID: CHEMBL4794), sodium channel protein type IX alpha subunit (CHEMBL_ID: CHEMBL4296) and renin (CHEMBL_ID: CHEMBL286) have the biggest ratio (> 8). Nevertheless, each of these targets still has at least 40 active and at least 30 inactive compounds.

### QSAR models

For each target, the average sensitivity, specificity and correct classification rate (CCR) were calculated over the 100 different models generated. The average values are 0.80 (± 0.15), 0.81 (± 0.16), 0.81 (± 0.07), respectively. Hence, these results show good overall performance of the QSAR models with an ability to predict both active and inactive compounds. The individual results are all available in Additional file 2. Our experience suggests that a good QSAR model should have a CCR greater than 0.7, therefore it is encouraging to see that 92% (505) of the models meet this condition.

**Fig. 3:**
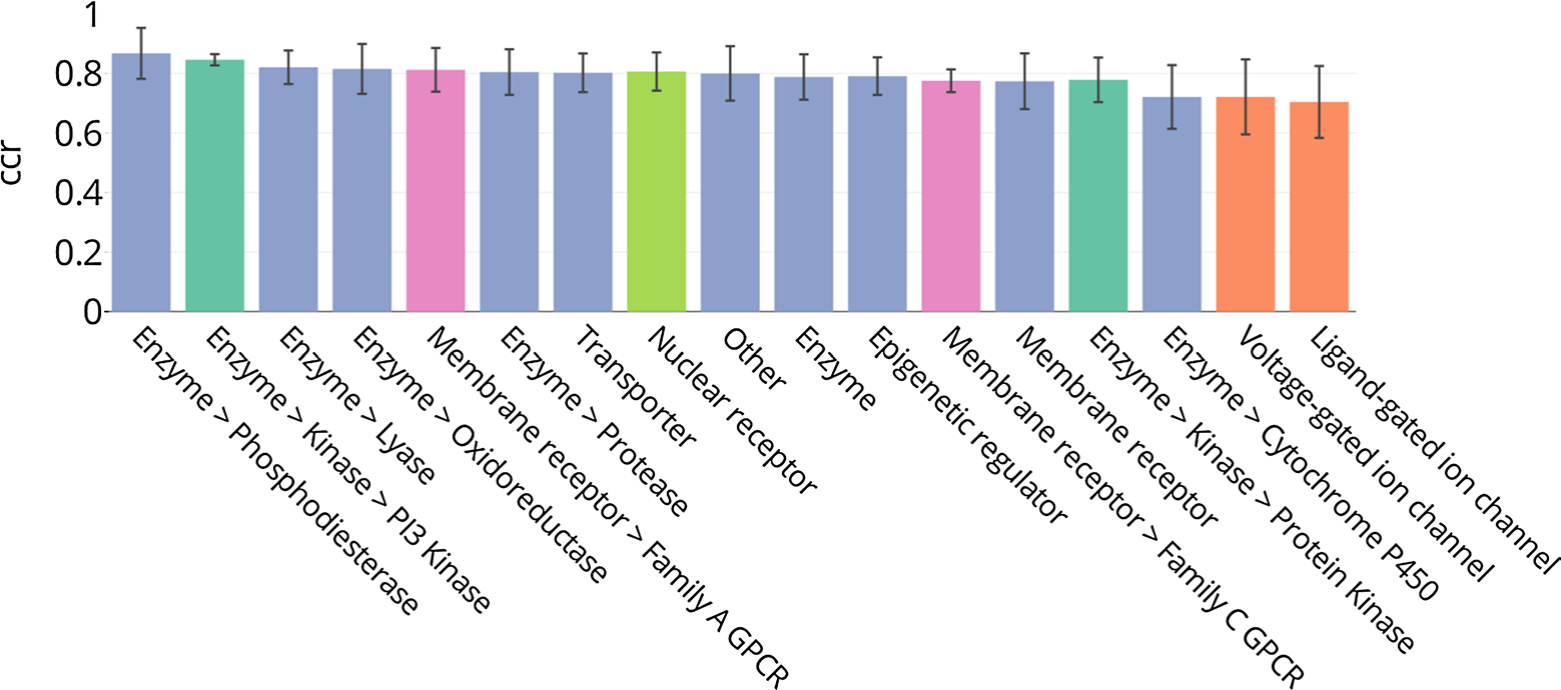
Mean CCR of the 550 QSAR models grouped by protein family

Figure 3 shows differences in the model predictivity for the different protein families as exemplified by the CCR. The models perform best on the phosphodiesterases and perform well (mean CCR > 0.7) for all the other protein families. However, the cytochrome P450 s and ion channels generally slightly underperform with significant variability in performance metrics across members of these families for the ion channels. For the cytochrome P450 s, the CCR values range from 0.59 to 0.89 and for the ion channels from 0.55 to 0.91 (Additional file 2). Therefore, despite these relatively low average CCR values, these two families show different behaviour regarding the prediction of active and inactive compounds. In particular, the ion channel models are good at predicting active compounds with 0.86 ± 0.2 and 0.93 ± 0.07 sensitivities for voltage-gated and ligand-gated ion channel families, respectively (Additional file 1: Figure S2). On the other hand, they demonstrate low predictivity for the inactive class with specificities of 0.62 ± 0.27 and 0.54 ± 0.22, respectively (Additional file 1: Figure S3). The cytochromes P450 exhibit the opposite behaviour with globally good specificity (0.84 ± 0.20) and relatively poor sensitivity (0.67 ± 0.27).

### Mondrian CP models

To ensure consistency, the same Random Forest algorithm and associated parameters were used in the MCP framework as for the QSAR models. The class assignment was done at different confidence levels (70, 80 and 90%) and all the individual results for different confidence levels are available in Additional file 3. The MCP results described here are for all the models built.

The MCP model performance was first assessed in term of validity. Firstly, 97.6%, 100% and 100% of the models were valid at 70%, 80% and 90% confidence level, respectively. Secondly, we looked at the validity for each class and in particular the number of models where the least represented class did not reach this criterion. Interestingly, it appears that a large majority fulfil the validity criteria. At the 70% confidence level, 90% of the models have their least represented class being valid, 97% at 80% confidence level and 99% at a confidence level of 90%. These results show that the MCP framework is particularly well suited for both the imbalanced and balanced data sets that are represented in our panel.

The analysis of the class assignment shows important differences with respect to the confidence level (Table 2). In particular, the number of compounds assigned to the ‘both’ class increases with the user-defined confidence level (as would be expected). It is on average less than 1% at 70% confidence, around 8% at 80% and more than 30% at 90%. This phenomenon is inherent to conformal predictors [18, 24, 41] and is also inversely correlated to the percentages of compounds assigned to the ‘empty’ class. At a 70% confidence level, conformal predictors tend to assign compounds to the ‘empty’ class because the *p* values are below the significance cut-off. If a higher confidence level is required, the cut-off is decreased and the compounds are then classified either in a single class (the correct or the incorrect one) or to the ‘both’ class.

**Table 2:** Fraction of compounds assigned in the ‘both’ and ‘empty’ prediction classes by the MCP models at different confidence levels

Confidence level	70%	80%	90%
‘Both’	0.01 (± 0.04)	0.08 (± 0.12)	0.32 (± 0.21)
‘Empty’	0.16 (± 0.08)	0.04 (± 0.05)	0.002 (± 0.009)

CP is often presented as a different approach to define the applicability domain (AD) of a model [18, 24, 25]. Indeed, it is reasonable to argue that a compound assigned to the ‘empty’ class is too dissimilar from the molecules in the model and so cannot be part of the AD. Our results show that, at lower confidence level, more compounds are assigned in the ‘empty’ class and therefore are left out of the AD. At higher confidence levels MCP is prone to maximise the number of ‘both’ classifications. Hence the predictions are neither correct nor incorrect but it becomes impossible to assess the AD.

**Fig. 4:**
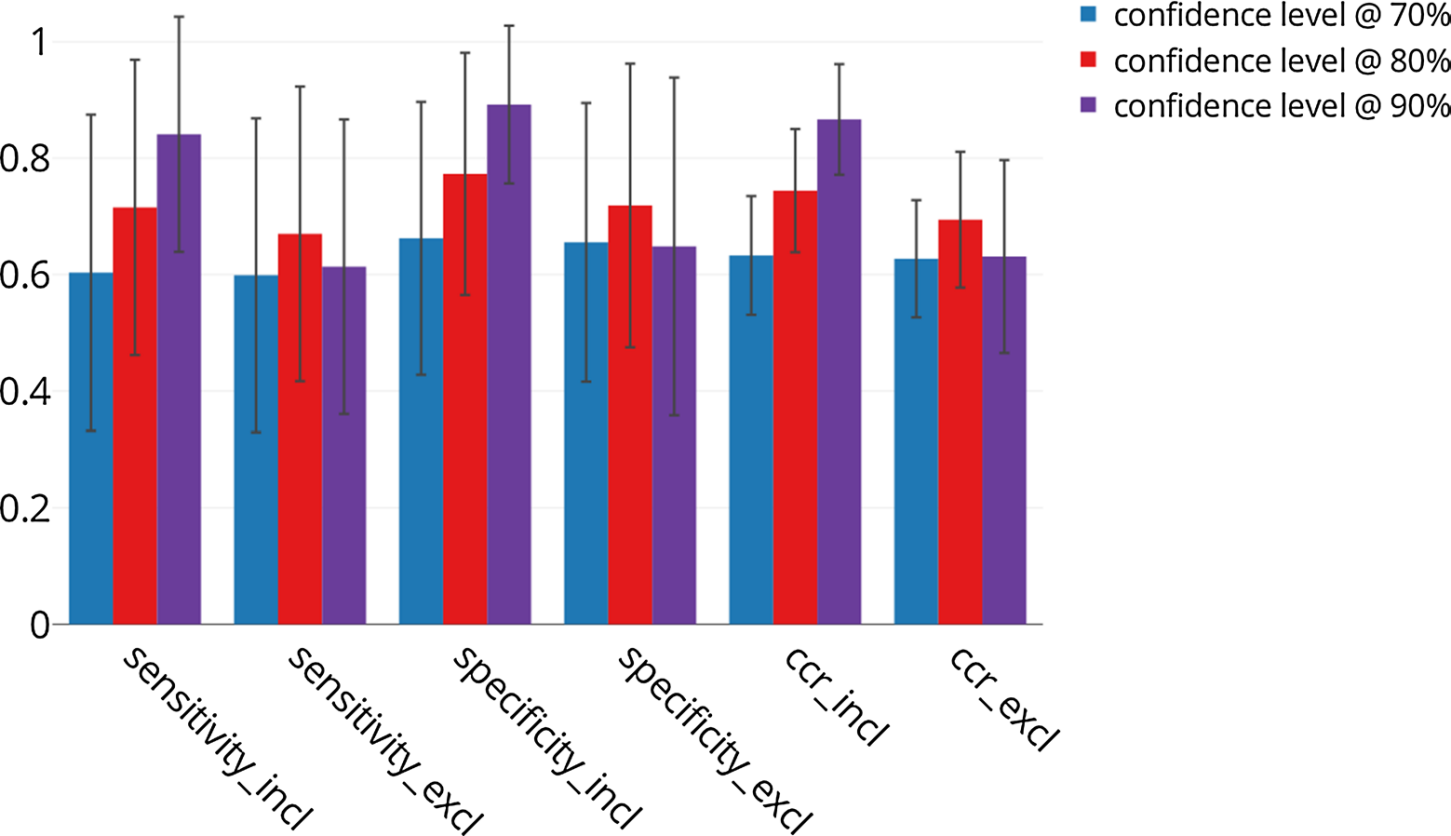
Overall sensitivity, specificity and CCR for the 550 conformal predictors at different confidence levels. Results show the performance according to whether the ‘both’ predictions are included or excluded from the calculation

The number of compounds predicted in the ‘both’ class might have a major impact on the performance assessment of the models, in particular when its proportion can exceed 30% as is the case for some of the models described here. This is why we opted to directly compare results according to whether this class is included or excluded in the performance metrics. Analysis of the global performance at 70%, 80% and 90% confidence levels highlights differences in predictive performance and is shown in Fig. 4.

When compounds predicted in the ‘both’ class are included, the sensitivity_incl, specificity_incl and ccr_incl metrics increase with the confidence level, from 0.74 (± 0.02) at 70% to 0.94 (± 0.02) at 90%, for the three metrics (Fig. 4). When the ‘both’ class is excluded from the metric calculation, very little difference is observed at 70% confidence level (Fig. 4). The lowest sensitivity_excl and specificity_excl are both observed at 90% with 0.63 (± 0.20) and 0.62 (± 0.20), respectively. The highest are obtained at 80% with 0.76 (± 0.11) for both metrics. Consequently, the values of the CCR follow a similar trend with 0.62 (± 0.19) at 90% and 0.76 (± 0.11) at 80% confidence level. The variability between the targets is particularly important at the 90% confidence level, as indicated by the standard error bars on the Fig. 4. For all the metrics, there is an increase in performance metrics at 80% confidence but they then decrease when the confidence is set too high (Fig. 4). This result needs to be compared with results in Table 2 that show a higher percentage of compounds in the ‘both’ class as the confidence level increases.

Once grouped by protein families and using the CCR metric for comparison, the results show, as for the overall results, that the family order is little affected by the omission of the ‘both’ class at 70% confidence level (Additional file 1: Figure S4). All protein families manage to pass the performance threshold of 0.7 in both conditions. At the 80% confidence level, the CCR values increase for each family including the ‘both’ prediction class but decrease, sometimes significantly, when they are excluded. Hence, the models for the ion channel families perform among the best in the first situation but their performance declines afterwards to reach levels similar to that observed for their QSAR counterparts. At the 90% confidence level the family performance increases when the ‘both’ prediction class is considered but, as for 80% confidence level, they decrease when it is removed. The phosphodiesterase family is the least affected by this phenomenon with a CCR that decreases by 0.17 (from 0.93 + 0.01 to 0.76 ± 0.12) while the ligand-gated ion channel model performance decreases significantly from 0.95 (± 0.02) to 0.47 (± 0.23). In comparison with the QSAR models, at this high confidence level, MCP models outperform QSAR but excluding the ‘both’ predictions, MCP returns a similar ordering of the protein families but with a lower CCR in all cases.

Therefore, it appears clear that the results of MCP are affected by the confidence level and is related to the compounds predicted as both active and inactive. At 70% confidence level, as shown in Table 2, these predictions are marginal and so have little effect. However, as the confidence increases the effect becomes more pronounced, with MCP assigning more and more compounds to the ‘both’ prediction class. The specific application may then become important. For example, a user wanting to select just a few compounds for a deep experimental analysis is more likely to use a high confidence and to consider only the compounds predicted as active. On the other hand, when prioritising compounds for a primary screen, molecules in the ‘both’ class might be included, excluding only the compounds predicted as inactive or in the ‘empty’ class. Hence, how to treat compounds that can be either active or inactive and which confidence level to use is tightly linked to the task the user wants to achieve. It is important to take into consideration that in the MCP framework, high confidence needs to be balanced against prediction certainty.

The effect of the number of compounds on the CCR was further investigated to see if it has an effect on the model performance. Our results suggest that when the compounds predicted in both classes are considered as correct, this parameter has little effect (Additional file 1: Figure S5 A, B and C). However, when excluding the compounds, we observed that some models with fewer compounds cannot maintain their performance in particular at the 80% and 90% confidence levels (Additional file 1: Figure S5 D, E and F). Hence, using MCP, we were able to generate good performing models for targets with few data points available when sacrificing on the interpretability of the results due to the compounds assigned in both classes. While the QSAR models are little affected by this parameter, we will see in the next section that unlike the MCP models, the ratio of active to inactive compounds does have an impact on their performance.

### Influence of the ratio of active to inactive compounds

**Fig. 5:**
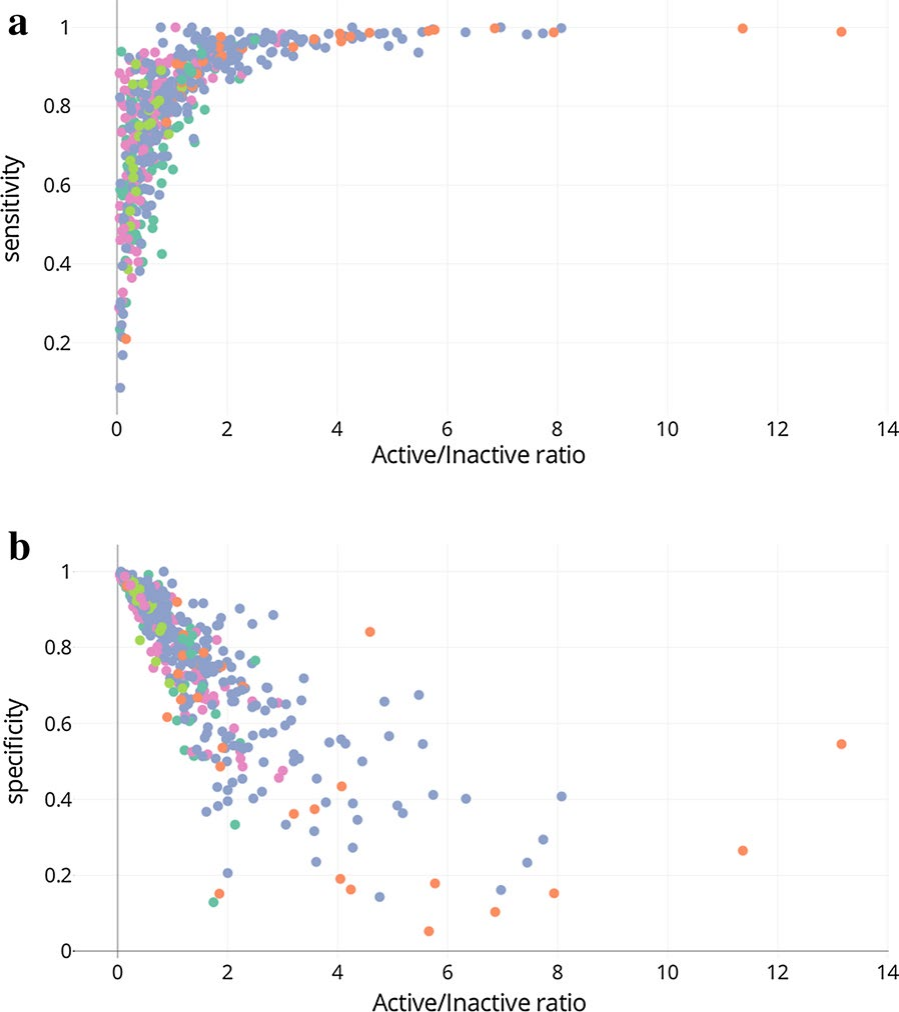
Sensitivity (**a**) and specificity (**b**) versus the ratio of active to inactive compounds for each QSAR models. Colours represent the protein families as described in the legend of the Fig. 3

The protein targets have different ratios of active and inactive compounds (Additional file 1: Figure S1) and this may have an influence on the model performance. Looking at the individual QSAR models, we observed that unbalanced data sets tend to result in predictions oriented toward one or the other class. Indeed, the models with the highest sensitivity are those with the highest ratio of active to inactive compounds (Fig. 5a) whereas those with the highest specificity have the lowest ratios (Fig. 5b). This is consistent with previous studies that have already demonstrated that when class sizes differ greatly, classifiers tend to favour the largest one leading to poor prediction for the minority class [24, 42–45].

Interestingly, the ratio seems to have less of an impact on MCP performance. Firstly when the ‘both’ prediction class is included and at each confidence level, there is no effect on the sensitivity (Additional file 1: Figure S6 A, B and C) or on the specificity (Additional file 1: Figure S7 A, B and C). However, when we exclude the ‘both’ class, there is much more variability in the results. The best illustration is at 90% confidence level where models having a low ratio can exhibit good or bad sensitivity/specificity (Additional file 1: Figure S6 D, E and F, and Additional file 1: Figure S7 D, E and F).

The two ion channel families delivered among the worst results using both QSAR and MCP. To try and understand why, we looked in detail at the individual models in these families. Several of the protein targets have either high or low active/inactive ratios that directly impact their performance. For the ion channels the most significant examples are the Vanilloid receptor (CHEMBL_ID: CHEMBL4794) with a ratio of 13 and the Voltage-gated N-type calcium channel alpha-1B subunit (CHEMBL_ID: CHEMBL4478) with a ratio of 0.16. The former target is involved in the nociception mechanism and many programmes have been initiated to develop potent antagonists that show activities better than nanomolar [46–49]. The latter suffers from an activity threshold of 6.5 compared with 5 for most of the ion channels as recommended by IDG, combined with activities mostly measured *in cellulo* leading to high IC50 values, resulting in a high percentage of compounds classified as inactive for this protein. The cytochrome P450 family, for which the models are less predictive mainly in QSAR, shows low active/inactive ratios (< 0.2) for half the proteins, indicating a high proportion of inactive compounds. The proteins in this family are often screened early in a drug discovery programme with the aim of specifically identifying compounds with low P450 inhibition and so it is not surprising to find many inactive compounds for these proteins in the scientific literature. Note that the use of balanced weights for the classes during the QSAR training results in limiting the ratio effect with a mean CCR of 0.76 (Fig. 3). Although a low or high ratio does not necessarily lead to a poor model, these examples show that discrepancies in the distribution of active and inactive compounds for these families are more likely affect the model predictivity, in particular for QSAR and MCP models excluding the ‘both’ prediction class. This suggests that the use of more balanced sets of active and inactive compounds using diffent thresholds could generate improved models.

However, this ratio alone does not always explain the model performance, in particular considering MCP where the ‘both’ class prediction is excluded. For example, the models corresponding to the targets Sodium channel protein type X alpha subunit (SCN10A, CHEMBL_ID: CHEMBL5451) and Vascular endothelial growth factor receptor 3 (VEGFR3, CHEMBL_ID: CHEMBL1955) have balanced active/inactive ratios of 1.08 and 1.02, respectively. However, at 80% confidence level, their sensitivity_excl and specificity_excl values indicate differences in the model’s ability to predict both active and inactive compounds in the correct single class. SCN10A and VEGFR3 have sensitivities of 0.80 and 0.41, and specificities of 0.88 and 0.38, respectively. In the case of SCN10A, when comparing the 11 actives present in the test set with the 13 actives in the calibration set, a median similarity of 0.51 was calculated using the Tanimoto coefficient (Additional file 1: Table S2). A similar comparison of the inactive compounds shows a median similarity of 0.5 between the 10 compounds in the test set and the 12 in the calibration set (Additional file 1: Table S3). In both cases, the compounds in the test set are thus similar enough to those of the calibration set to allow the Mondrian conformal predictor to attribute high *p* values to the right class which allows good assignments when compared to the required confidence level. In contrast, for the 13 active compounds present in the VEGFR3 test set, the median similarity is only 0.33 compared to the 15 compounds in the calibration set (Additional file 1: Table S4), and 0.29 for the 12 inactive compounds in the test set compared to 14 in the calibration set (Additional file 1: Table S5).

Comparing these results with those obtained when the ‘both’ class is included, both targets have equivalent high sensitivities and specificities (SCN10A: 0.80 and 0.88, and VEGFR3: 0.89 and 0.88, respectively). In the case of VEGFR3, this shows that even if the molecules in the test set are dissimilar to those in the calibration set, they may have molecular features present in both active and inactive compounds which means that the conformal predictor cannot determine to which class a predicted compound should be assigned.

### Comparison of the QSAR and Mondrian CP models

**Fig. 6:**
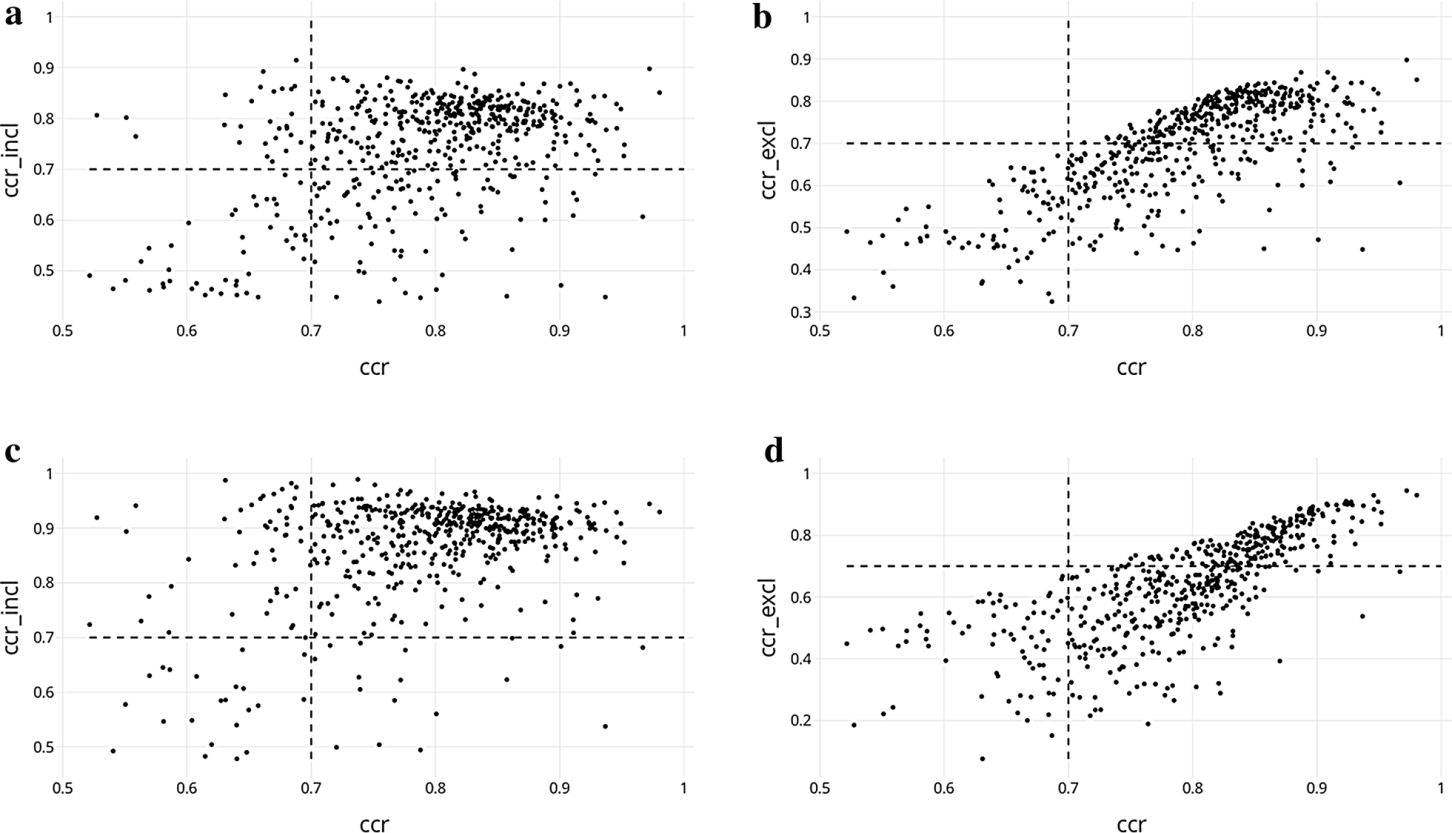
CCR comparison between results of QSAR and MCP models at 80% (**a**, **b**), and 90% (**c**, **d**). In **a**, **c** The ‘both’ class prediction is included for model evaluation while it is left-out in (**b**, **d**). The targets are divided in four quadrans depending on whether they have good results for both MCP and QSAR (upper-right), either MCP (upper-left) or QSAR (bottom-right), or none of them (bottom-left)

The objective of this section is not to conclude that one or the other approach outperforms the other but rather to investigate whether the results from QSAR and MCP differ for different protein targets. As we have seen in the previous sections, QSAR and MCP are affected differently by the ratio of active to inactive compounds. Additionally, we did a direct comparison of the model results at 80% confidence level because as shown earlier, it offers the best overall distribution of ‘both’ and ‘empty’ (Table 2) and therefore a balanced result no matter how the ‘both’ prediction class is used. The comparison was also made at 90% confidence level because it gives the best performance for MCP when the compounds assigned in the ‘both’ class are considered. For each target model, the CCR values for the QSAR and MCP models were plotted according to whether or not the ‘both’ class was used in the MCP results (Fig. 6). A CCR of 0.7 was used to define the minimum limit of performance required to consider a model as “good”.

First, comparing QSAR with MCP and including the ‘both’ class (Fig. 6a), it appears that 505 (92%) of the targets have good performing models for both approaches. No target shows poor results with both modelling methods, nor does QSAR outperform MCP. However, 45 (8%) targets have a MCP model that outperforms their corresponding QSAR model (Table 3). Therefore, it seems that MCP is more likely to produce a useful predictive model (505 QSAR models with a CCR ≥ 0.7 compared with 550 (100% of the models) for the conformal predictors). Using a confidence level of 90% and including the “both” compounds confirms this advantage of MCP over QSAR, for reasons we have already outlined (Fig. 6c).

**Table 3:** Classification of the targets according to their QSAR and MCP model performances

	Confidence level (%)	Poor QSAR/poor MCP	Good QSAR/poor MCP	Poor QSAR/good MCP	Good QSAR/good MCP
‘Both’ included	80	0	0	45	505
‘Both’ excluded		39	67	6	438
‘Both’ included	90	0	0	45	505
‘Both’ excluded		44	298	1	207

However, when excluding the compounds classified in the ‘both’ prediction class, the results of MCP at 80% confidence become more similar to those of QSAR (Fig. 6b). The proportion of targets with good MCP and QSAR models remains high with 438 (80%), but 67 (12%) now have only a good QSAR model whilst 6 (1%) have only a good MCP model (Table 3). Consequently, there are now 39 targets (7%) for which neither MCP nor QSAR were able to provide a good model. At 90% confidence level, only 38% of the protein targets (208) have a poor conformal predictor while 505 still have a good QSAR one (Fig. 6d).

Overall, the exclusion of the ‘both’ prediction class gives MCP lower but comparable performance to the QSAR models and this results in a better correlation between MCP and QSAR (Additional file 1: Figure S8). Moreover, as expected none of the targets sees its MCP-related model performance improved when excluding the compounds assigned in both classes. Hence, comparing the two MCP approaches at two different confidence level, it appears that (as pointed out previously), the use of the ‘both’ class gives better overall performance but ignoring it significantly decreases the conformal predictor performance in particular for high confidence levels. It confirms that the user will ultimately need to decide depending on the needs of the specific application.

**Fig. 7:**
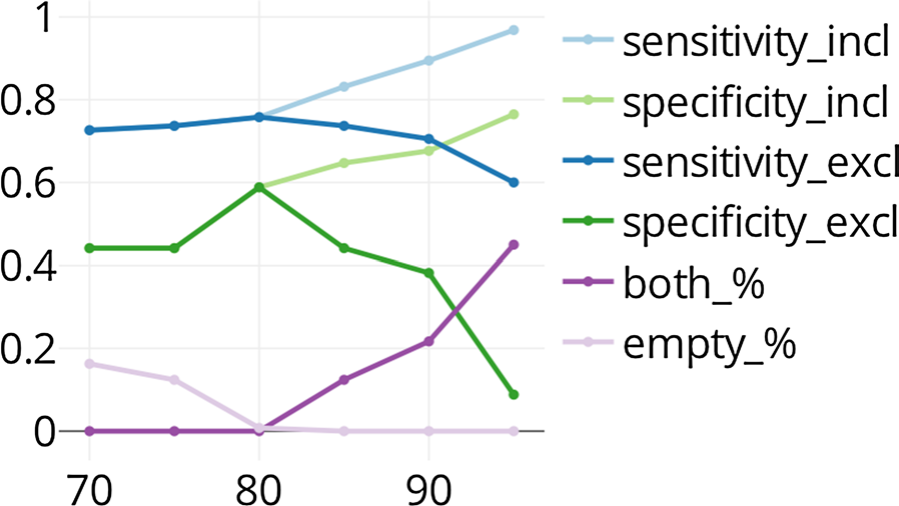
Evolution of the MCP performance depending on the confidence level for hERG

To give an example of comparison between the two techniques, we focus here on the case of the ion channel hERG (CHEMBL_ID: CHEMBL240). This protein is a potassium channel located in the heart and provides an essential contribution to the repolarisation of the cardiac action potential. Mutation or inhibition of this target can induce life-threatening arrhythmia [50]. It is a protein commonly screened to assess such risks. The hERG QSAR model shows good performance with sensitivity and specificity values of 0.83 and 0.81 demonstrating a particularly good ability to identify the active class, i.e. potentially toxic compounds (Additional file 2). The corresponding Mondrian conformal predictor manages to reach similar performance at 80% confidence level whether or not the ‘both’ prediction class is included with values around 0.8 for the two metrics respectively (Additional file 3). However, by increasing the confidence, these metrics improve and at 90%, we reach 0.92 and 0.92, respectively (Fig. 7). However, this performance is only obtained by sacrificing some interpretability of the results as 27% of the compounds are predicted in the ‘both’ class. Removing them strongly decreases the advantage of MCP over QSAR with performance values of 0.66 and 0.65. Note that decreasing the MCP confidence level does not give better predictivity. Therefore, given the nature of the target, it might seem judicious to use the highest confidence even if it leads to 30% uncertain predictions. If, however, one wanted to focus just on the compounds predicted as active, it might be worth lowering the confidence or alternatively to use the QSAR model directly.

### Temporal validation

No matter how good the internal validation, the biggest challenge of any machine learning approach is in correctly predicting prospective data. For both QSAR and MCP, it can be particularly challenging to predict compounds that are structurally very different from the chemical space in which they were trained. To further assess the performance of our models, temporal validation was applied. Relying on the assumption that new molecular scaffolds are published every year, this kind of validation consists of the prediction of data published a posteriori of the training data. Because ChEMBL extracts data on an on-going basis for each release, it is possible to use distinct versions of the ChEMBL database to generate data sets temporally different which are therefore likely to differ in terms of chemical space coverage.

Using the latest ChEMBL release (version 24), new experimental data were extracted for 296 targets and evaluated on their corresponding QSAR and MCP models. With an average number of 6.8 new compounds per target, it was not meaningful to calculate the performance of the individual model or even for the protein families. Hence, the method performances were grouped and evaluated globally.

The QSAR models on the temporal set return a sensitivity, specificity and CCR of 0.61, 0.80 and 0.71, respectively. Therefore, the prediction of inactive compounds remains in the range of what was observed globally in the internal validation (on 550 targets). The sensitivity remains good but the gap with the specificity suggests that some active compounds in ChEMBL_24 might be more dissimilar to those in the ChEMBL_23 set. Consequently, the CCR decreases compared to what was observed with the training set but remains good.

**Fig. 8:**
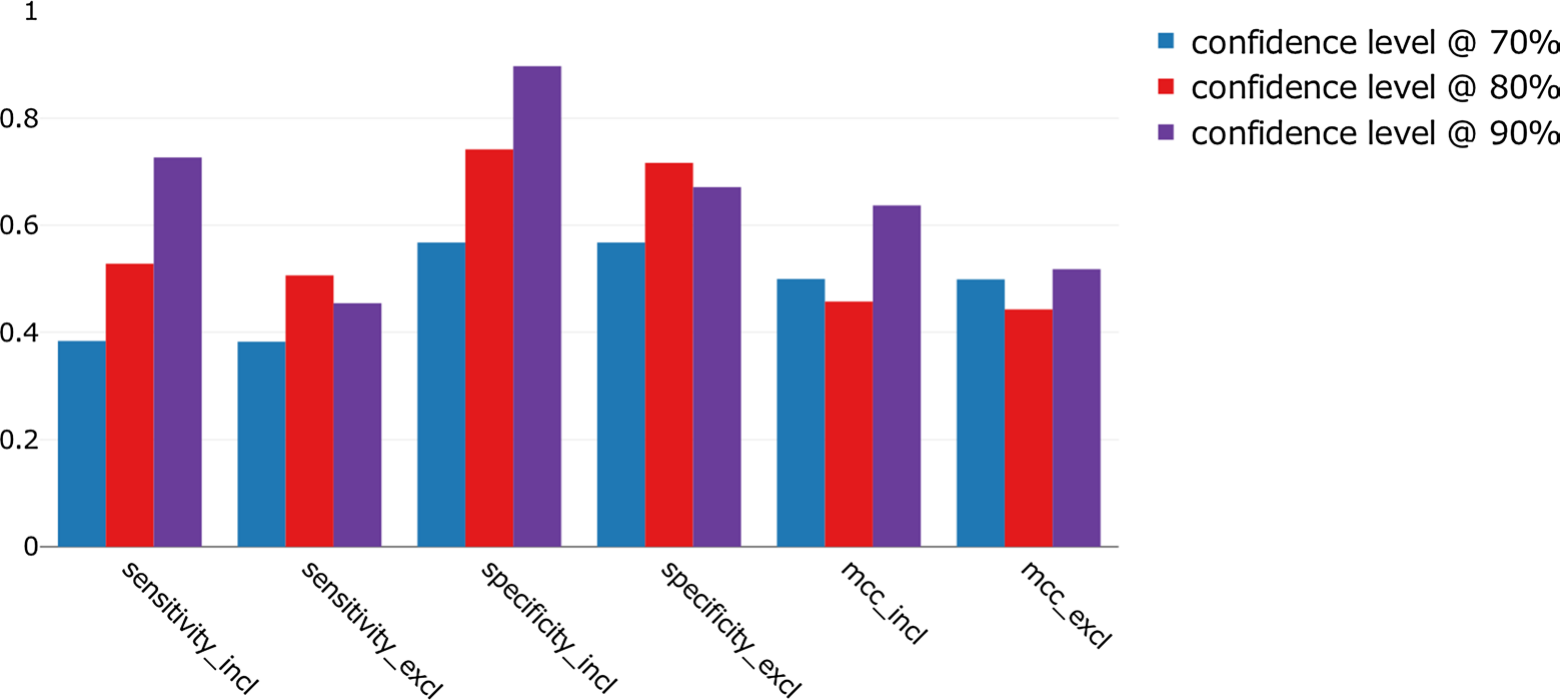
Performance of the MCP models on the temporal validation set at different confidence levels. The results show the performance according to whether the ‘both’ predictions are included or excluded from the calculation

The MCP results, as we have already seen, vary depending on the confidence level used and the consideration of the ‘both’ prediction class. First, sensitivity and specificity evolve similarly with the confidence level (Fig. 8). Taking into account the ‘both’ class predictions, the sensitivity_incl increases from 0.41 at 70%, to 0.63 at 80% and 0.85 at 90%, and the specificity_incl values are 0.45, 0.67 and 0.87, respectively. However, excluding the ‘both’ class predictions, both metrics increase as the confidence level increases from 70% to 80%, from 0.41 to 0.54 and from 0.44 to 0.60, respectively. Then, these values decrease at 90% to 0.42 for the sensitivity and 0.44 for the specificity. This is due to the proportion of the ‘both’ class that reaches 8% at 90% confidence level while it is below 1% when the confidence is lower (Additional file 1: Figure S9). The CCR is also affected whether or not the ‘both’ class predictions are considered when a confidence level of 90% is used. At this level, the CCR for the models including the ‘both’ prediction class reaches 0.86 compared with 0.43 when it is excluded. The greater number of compounds assigned to the ‘both’ prediction class at this confidence level results in globally better predictivity of the models (Table 4).

**Table 4:** Comparison of the results obtained for the internal and the temporal validation for the QSAR and the MCP models built on ChEMBL_23, considering the 296 protein targets shared by ChEMBL_23 and ChEMBL_24

Method	Prediction set	Model targets	Confidence level (%)	Sensitivity	Specificity	CCR
QSAR	ChEMBL_23	296		0.78 (± 0.15)	0.84 (± 0.14)	0.81 (± 0.07)
QSAR	ChEMBL_24	296		0.61	0.80	0.71
MCP_incl	ChEMBL_23	296	70	0.73 (± 0.03)	0.73 (± 0.03)	0.73 (± 0.02)
MCP_incl	ChEMBL_23	296	80	0.84 (± 0.02)	0.84 (± 0.03)	0.84 (± 0.02)
MCP_incl	ChEMBL_23	296	90	0.94 (± 0.02)	0.93 (± 0.02)	0.93 (± 0.02)
MCP_incl	ChEMBL_24	296	70	0.41	0.45	0.43
MCP_incl	ChEMBL_24	296	80	0.63	0.67	0.65
MCP_incl	ChEMBL_24	296	90	0.85	0.87	0.86
MCP_excl	ChEMBL_23	296	70	0.72 (± 0.04)	0.73 (± 0.03)	0.73 (± 0.02)
MCP_excl	ChEMBL_23	296	80	0.77 (± 0.11)	0.77 (± 0.11)	0.77 (± 0.11)
MCP_excl	ChEMBL_23	296	90	0.65 (± 0.19)	0.63 (± 0.20)	0.64 (± 0.19)
MCP_excl	ChEMBL_24	296	70	0.41	0.44	0.42
MCP_excl	ChEMBL_24	296	80	0.54	0.60	0.52
MCP_excl	ChEMBL_24	296	90	0.42	0.44	0.43

As already described, the percentage of compounds assigned in the ‘empty class’ is inversely correlated to the confidence level [18]. Hence, it appears that up to 43% of the predicted molecules are too dissimilar from the molecules in the training set to be predicted at the 70% confidence level, 27% at 80% and 13% at 90% (Additional file 1: Figure S10). Therefore, the molecules introduced in ChEMBL_24 do not differ significantly from those in ChEMBL_23 for the same set of targets. This explains why the results obtain in the temporal validation are close to those of the internal validation.

Finally, in light of the results presented, is one modelling approach really better than the other? This question cannot be answered with a simple yes or no due not only to the different approaches used to build the models but also because it depends on the circumstances in which MCP or QSAR are to be applied. By definition, QSAR model always makes a prediction. Even if some compounds are outside the applicability domain, there is no alternative for this method but to assign a prediction to the correct or the incorrect class. With only two possibilities, there is only a one in two chance for the model to be right (or wrong). Consequently, both the number of correct and incorrect predictions can be increased theoretically in an equal way which is why the sensitivity and specificity are greater for the temporal validation of the QSAR models. To illustrate this statement, the confusion matrices of both QSAR and MCP with an 80% confidence level are compared (Table 5).

**Table 5:**
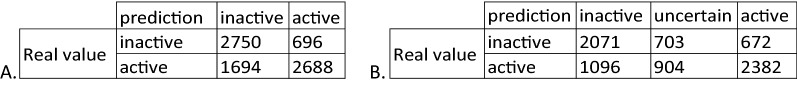
Confusion matrix for the prediction of ChEMBL_24 compounds using (A) QSAR, or (B) MCP with an 80% confidence level

For MCP, the uncertain class regroups compounds assigned either in the ‘both’ or in the ‘empty’ prediction classes

**Fig. 9:**
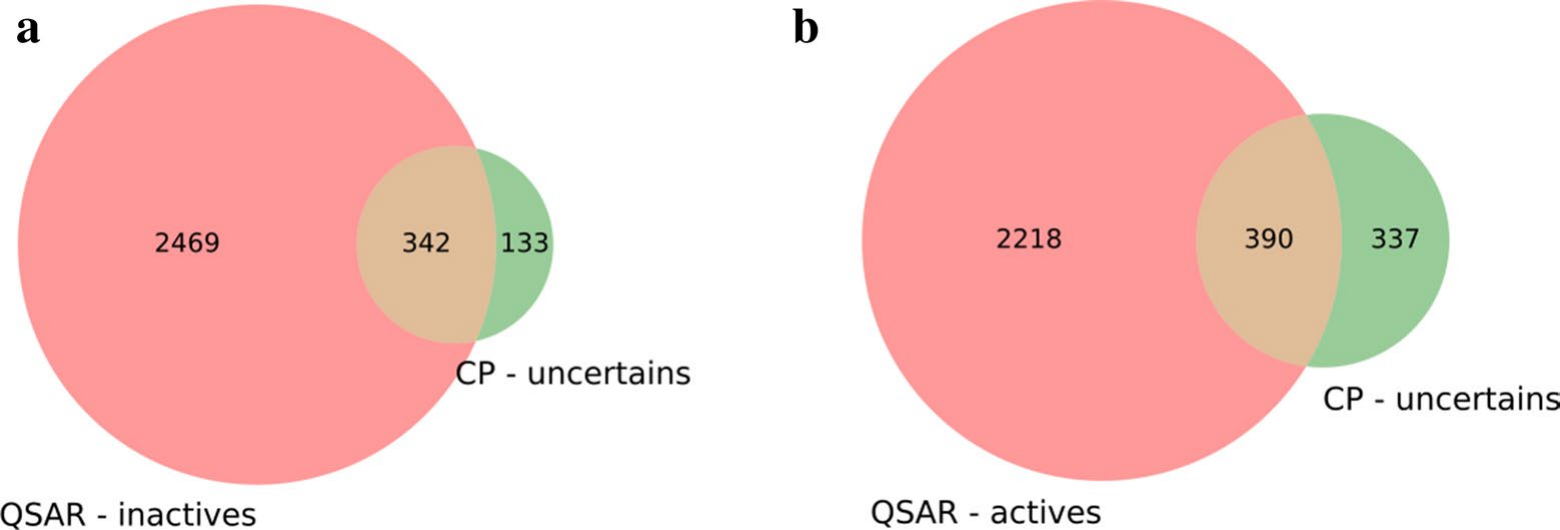
Comparison of the compound assignments in the uncertain class for MCP (at 80% confidence level) with QSAR for **a** the inactive and **b** the active compounds. The pink set represents the molecules (active or inactive) that are correctly predicted by QSAR, the green set represents the uncertain predictions from MCP and the brown set is the intersection between the sets, that is to say, the molecules predicted as uncertain by MCP but correctly predicted by QSAR

As already observed when we compared the performance metrics, the number of correct predictions is systematically greater with QSAR, and so too is the number of incorrect predictions. For MCP, the uncertain predictions, that include compounds assigned either to the ‘both’ or to the ‘empty’ prediction classes, result in a decrease in the number of correct predictions as well as the incorrect ones. Ignoring these predictions allows one to improve the overall predictivity. However, it can be problematic if a classification needs to be determined for all the molecules in the set. Indeed, in some cases MCP returned uncertain predictions, whereas QSAR was able to correctly classify the majority of them. For 703 inactive compounds of ChEMBL_24 in the uncertain category, 79% are correctly classified by QSAR (Fig. 9a), and for 904 active compounds, the proportion is 45% (Fig. 9b). Nevertheless, it is crucial to bear in mind that unlike QSAR, MCP associates a confidence score on the predictions assigned active or inactive. Hence it can be concluded that by associating a confidence to its predictions MCP offers the advantage of increased certainty in the prediction albeit at the expense of providing predictions on fewer compounds than QSAR.

### Temporal ChEMBL release model improvement

Considering the good performance of both the QSAR and MCP models in the temporal validation, the effect of the temporal gap between the data used to build the models and the data used in the prospective validation was investigated. Using version 22 of the ChEMBL database (ChEMBL_22), QSAR and MCP models were created using the same protocol as before. Models for 515 human targets were built. This is fewer than for ChEMBL_23 as insufficient data were available to build models using our defined criteria. Internal validation showed similar performance compared to models built on ChEMBL_23 data (data not shown) but it was of interest to assess the temporal predictivity of the models using ChEMBL_24. The comparison was performed on the 282 targets shared between ChEMBL_22 and ChEMBL_23 and for which there were new data in ChEMBL_24. The metrics were recalculated on this retrained number of targets for ChEMBL_23 models and the overall results of the comparison are presented in Table 6. MCP results were calculated at 80% confidence level only because, as observed in the previous section, this is the confidence level that offers the best balance between ‘empty’ and ‘both’ prediction classes for MCP.

**Table 6:** Performance of the models built on ChEMBL_22 and ChEMBL_23 data

Method	Model data	Prediction set	Model targets	Sensitivity	Specificity	CCR
QSAR	ChEMBL_23	ChEMBL_24	282	0.63	0.80	0.71
MCP_incl		ChEMBL_24	282	0.63	0.67	0.65
MCP_excl		ChEMBL_24	282	0.56	0.61	0.58
QSAR	ChEMBL_22	ChEMBL_23	282	0.64	0.84	0.74
		ChEMBL_24	282	0.60	0.81	0.71
MCP_incl		ChEMBL_23	282	0.61	0.72	0.66
		ChEMBL_24	282	0.61	0.67	0.64
MCP_excl		ChEMBL_23	282	0.56	0.68	0.62
		ChEMBL_24	282	0.54	0.61	0.57

MCP model results are given at 80% confidence level. MCP_incl and MCP_excl indicate the ‘both’ prediction class was included in the result calculation or was ignored, respectively

Globally, the prediction of ChEMBL_24 for both QSAR and MCP models improves slightly between ChEMBL_22 and ChEMBL_23 for both active compounds in particular. Therefore, it seems that the ChEMBL_23 models benefit from the influx of data. Both QSAR and MCP with a 80% confidence level are improved although QSAR models perform better. Note that as expected the results from the ChEMBL_22 models show that it is more difficult to predict data generated further in time, in particular for the inactive compounds.

## Conclusion

This manuscript has presented a detailed comparison between QSAR and MCP modelling methods when applied to a large data set of up to 550 human protein targets extracted from several versions of the ChEMBL database. The overall results demonstrate that both approaches can provide good predictive performance. Nevertheless, noticeable differences were observed for some targets. Whereas for the majority of targets MCP outperforms QSAR, there are a few examples that demonstrate the contrary. The influence of the ‘both’ prediction class is also a critical factor to take into account when applying the models in a research environment. It was also demonstrated that the degree of molecular similarity between the training, calibration and test sets has a major impact on the MCP results.

Using consecutive releases of the ChEMBL database, the robustness of the models was assessed using temporal validation. Although most models remain at an acceptable level of performance, a small decrease in the predictivity is seen, as expected. In the general case, the two approaches are very similar but MCP does provide a confidence value that is missing from traditional QSAR approaches and which can be a potentially useful piece of information to help with decision making in the context of practical drug discovery applications.

## Additional files

**Additional file 1.r**  Contains additional figures and tables supporting the work published in this paper.

**Additional file 2.**  Performance metric for the 550 QSAR models.

**Additional file 3.**  Performance metric for the 550 MCP models

## Abbreviations

AD: applicability domain
CCR: correct classification rate
MCP: mondrian conformal prediction
IDG: Illuminating the Druggable Genome
QSAR: quantitative structure–activity relationship
RF: random forests
