# Supplementary material for: Large scale comparison of QSAR and conformal prediction methods and their applications in drug discovery
Source: J Cheminform. 2019 Jan 10;11:4. doi: 10.1186/s13321-018-0325-4 (PMC6690068; doi:10.1186/s13321-018-0325-4)
Supplement: Supplementary file 1 — Additional file 1.r Contains additional figures and tables supporting the work published in this paper. [file 13321_2018_325_MOESM1_ESM.docx]

Comparative study of QSAR versus conformal prediction applied on ChEMBL data

Nicolas Bosc, Francis Atkinson, A. Patrícia Bento, Eloy Felix, Anna Gaulton, Fiona Hunter, Prudence Mutowo, Anne Hersey and Andrew R. Leach

Chemogenomics team, EMBL-EBI, Wellcome Genome Campus, Hinxton, Cambridge, CB10 1SD

Corresponding author: arl@ebi.ac.uk


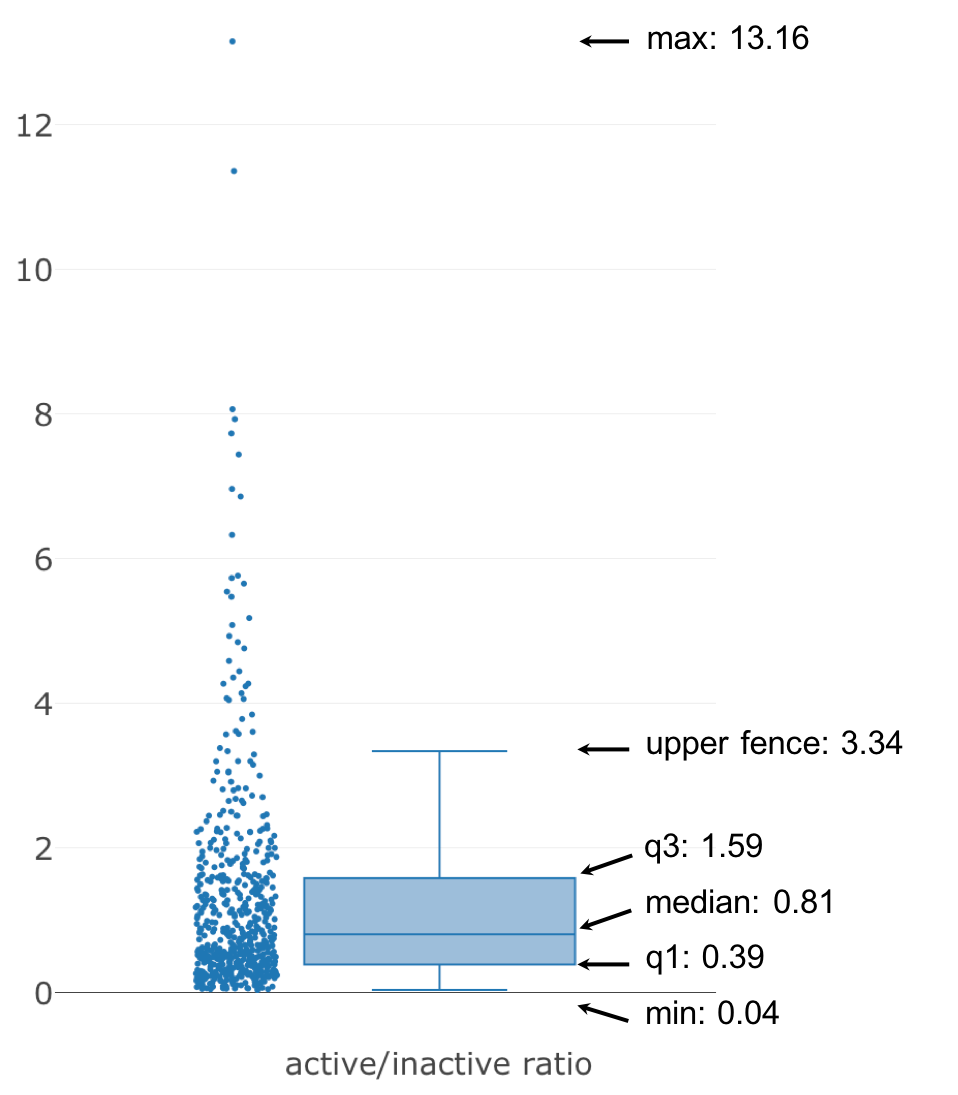


Figure S1: Active/inactive ratio for the 550 human targets selection for modelling


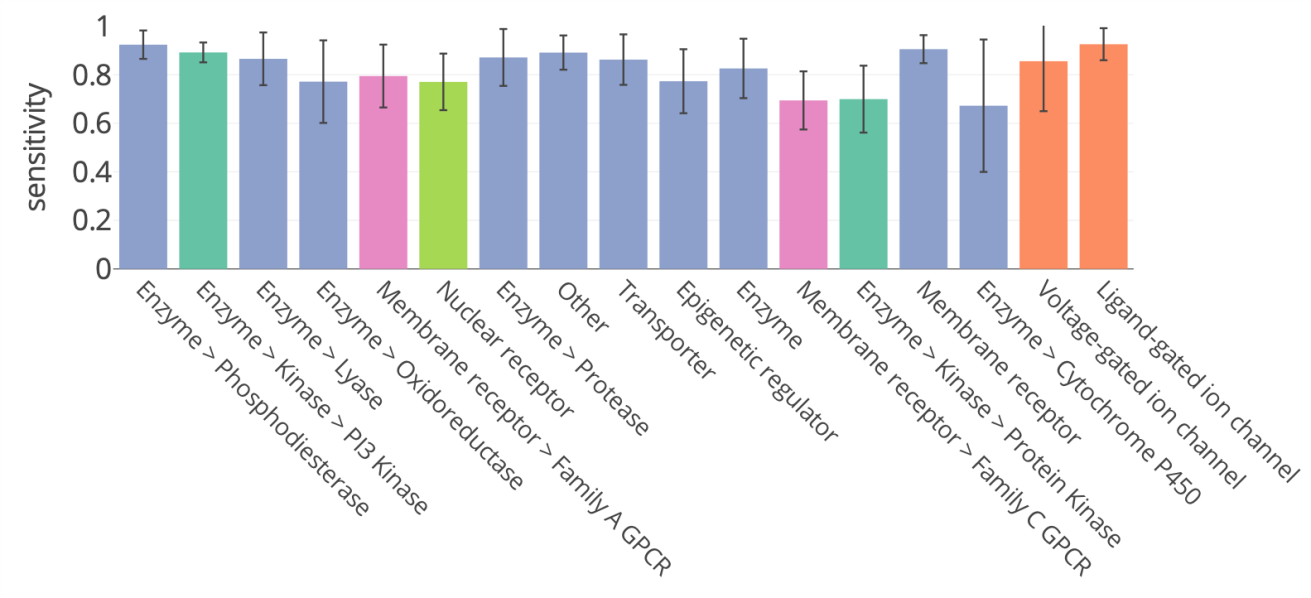


Figure S2: Mean sensitivity of the QSAR models grouped by protein families


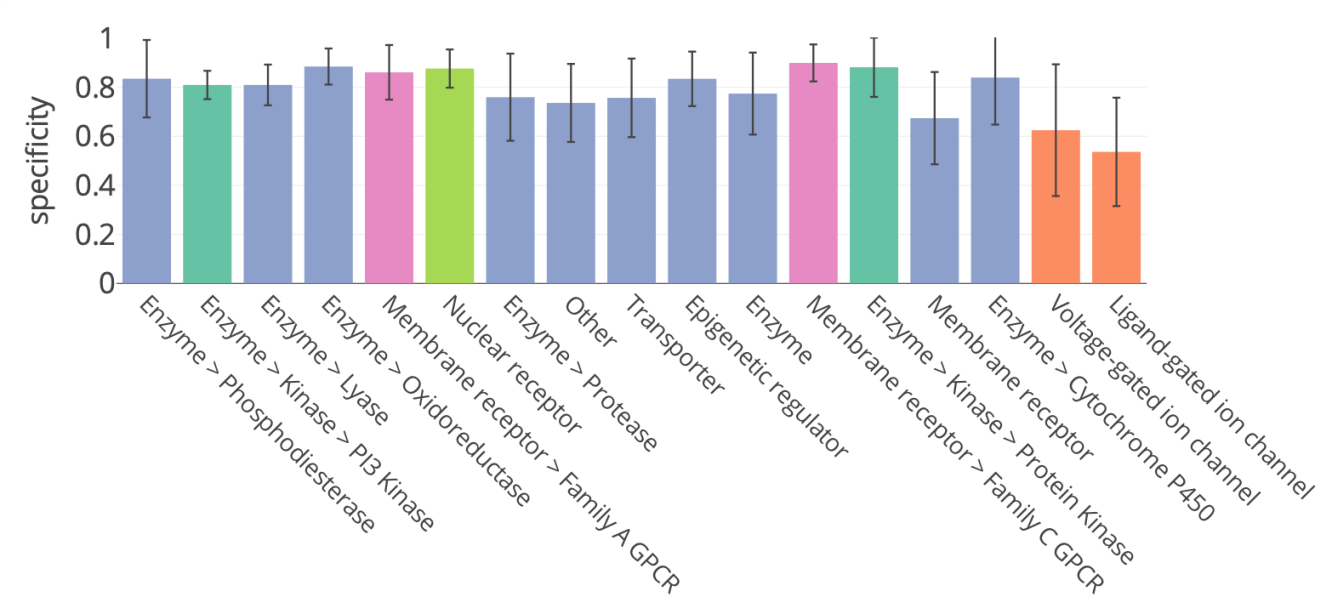


Figure S3: Mean specificity of the QSAR models grouped by protein families


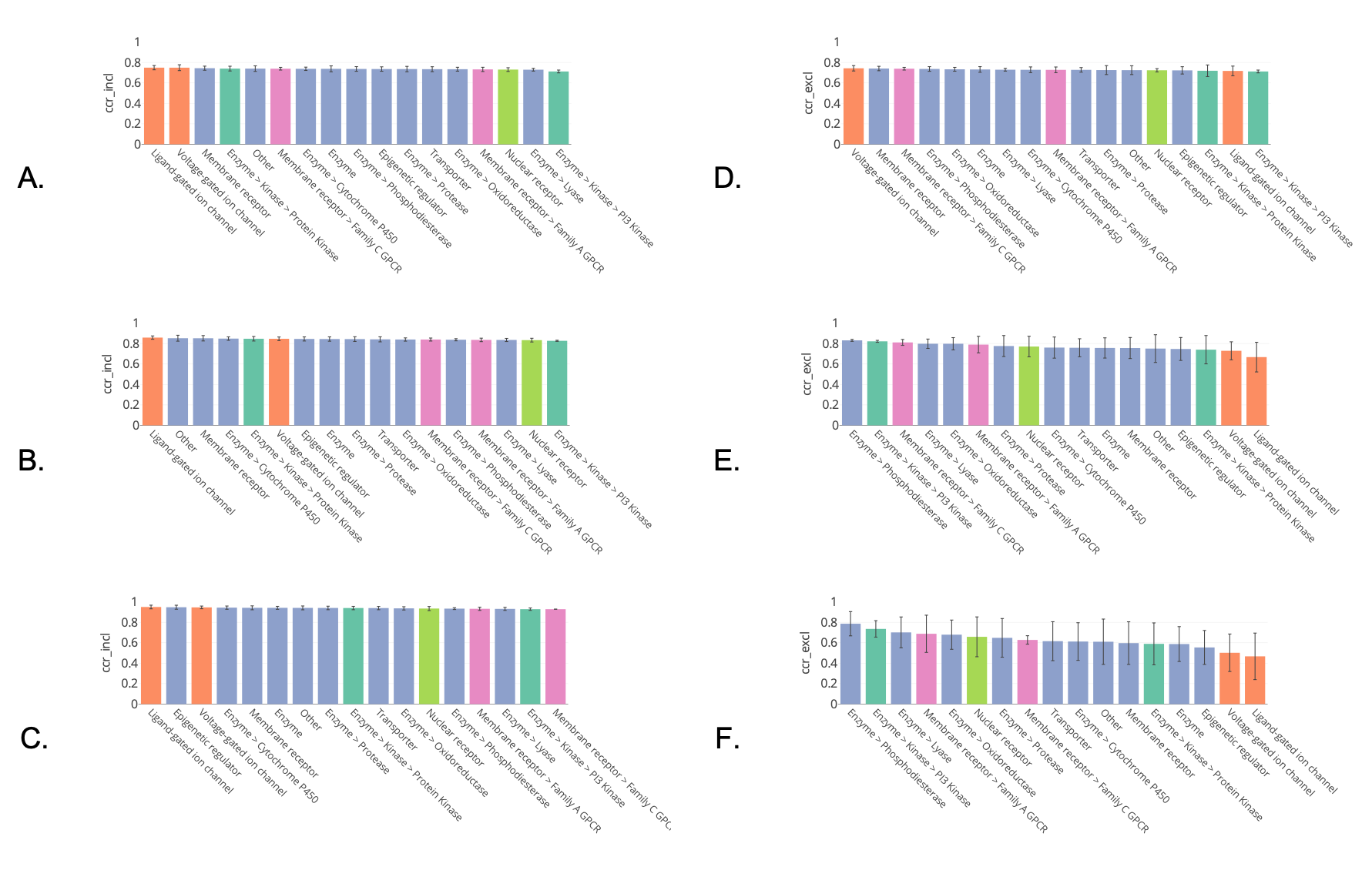


Figure S4: Mean CCR of the models grouped by protein families at 70%, (A) and (C), 80%, (B) and (D), and 90% (C) and (F), confidence levels, and depending on whether the ‘both’ class predictions are included (A), (B) and (C) or not (D), (E), (F)


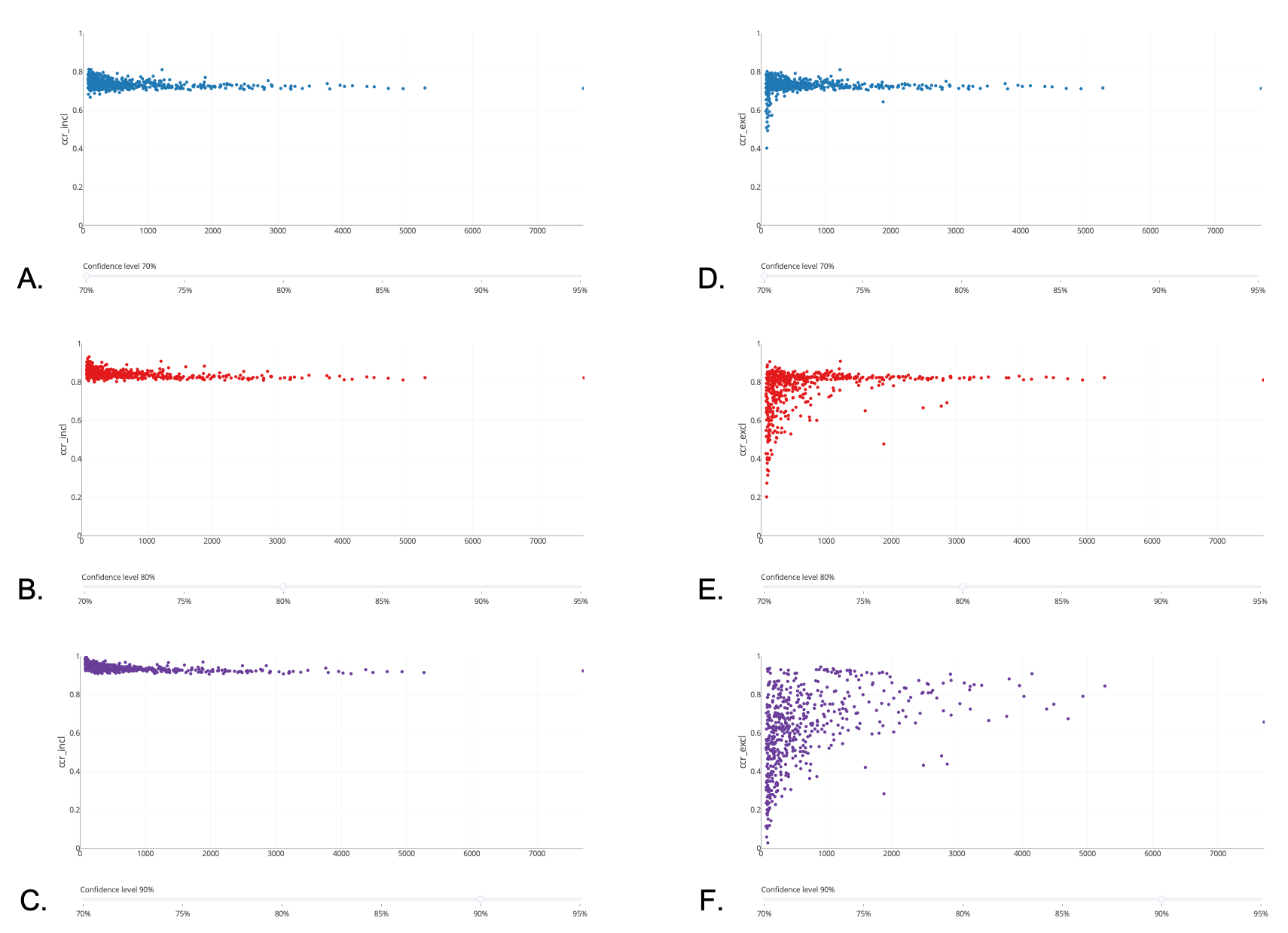


Figure S5:Global performance of the Mondrian conformal predictors depending on the total number of compounds in the data set, when the ‘both’ prediction class is included in the results ((A), (B) and (C)) or when it is excluded ((D), (E) and (F)). Panels (A), (B) and (C) show the CCR when at 70%, 80% and 90% confidence levels, respectively. Panels (D), (E) and (F) show the CCR at 70%, 80% and 90% confidence levels, respectively.


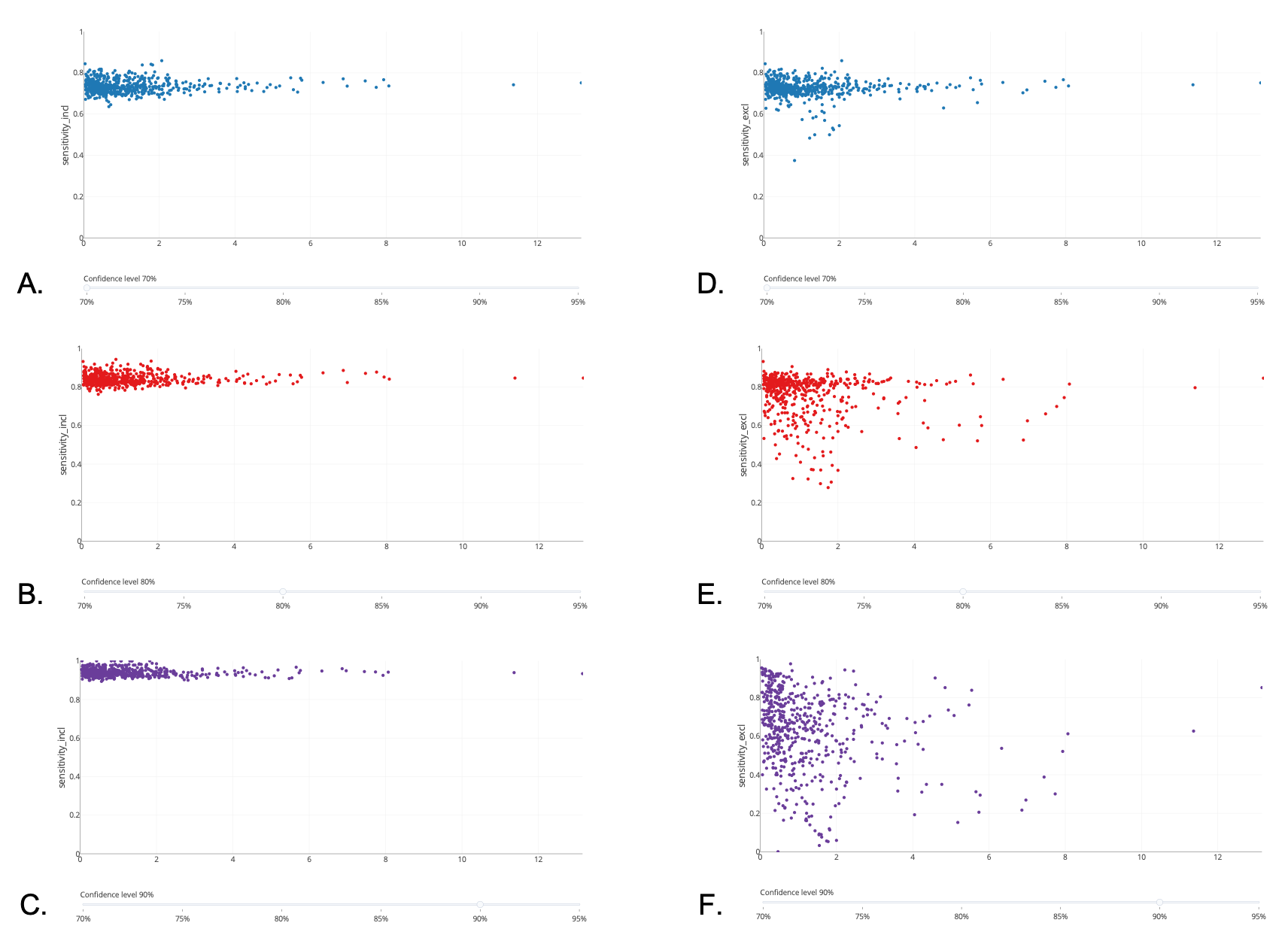


Figure S6: Sensitivity of the Mondrian conformal predictors depending on the ratio of active and inactive compounds whether ((A), (B) and (C)) or not ((D), (E) and (F)) the ‘both’ prediction class is included in the results. Panels (A), (B) and (C) show the sensitivity when at 70%, 80% and 90% confidence levels, respectively. Panels (D), (E) and (F) show the sensitivity at 70%, 80% and 90% confidence levels, respectively.


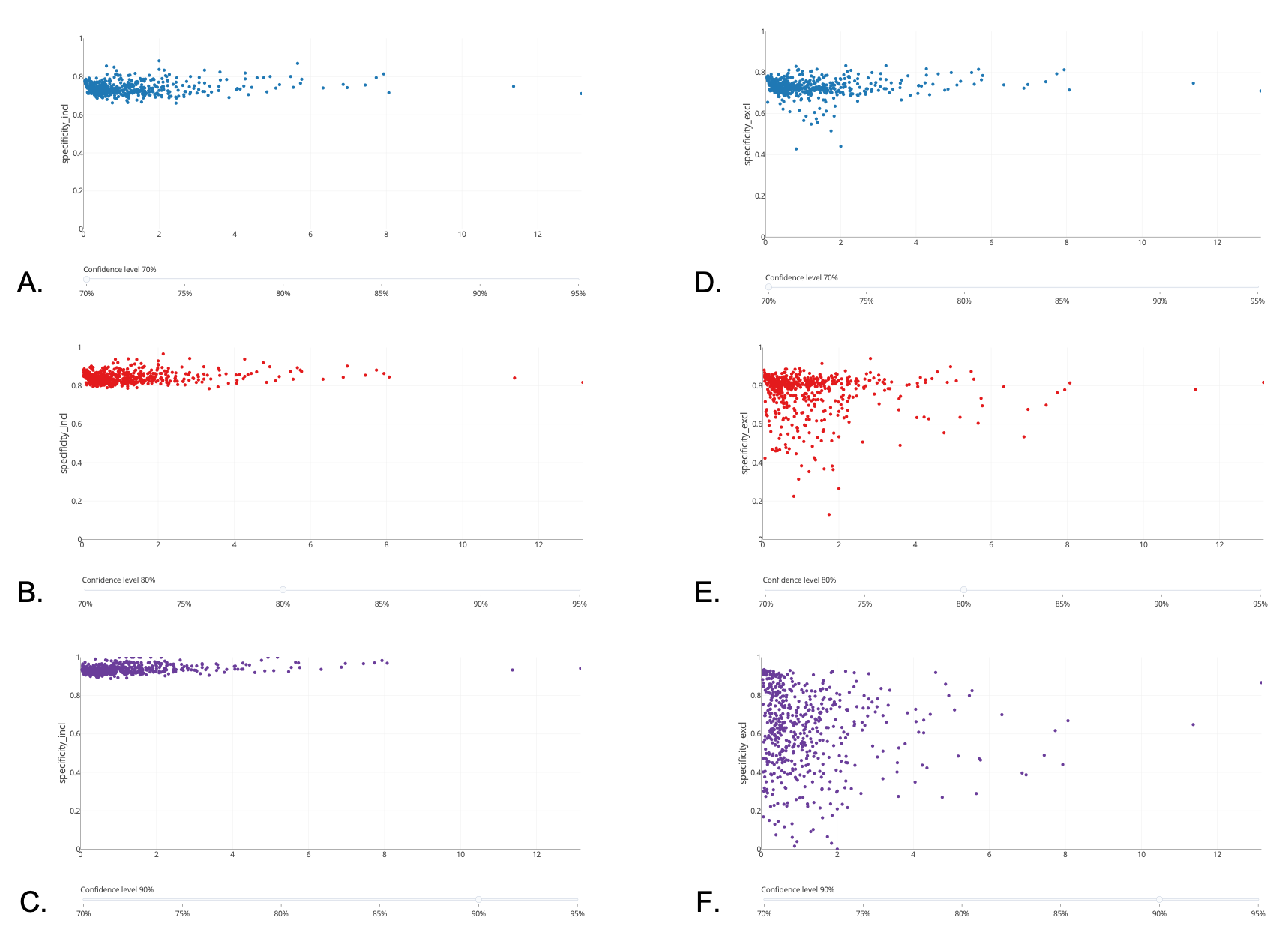


Figure S7: Specificity of the Mondrian conformal predictors depending on the ratio of active and inactive compounds whether ((A), (B) and (C)) or not ((D), (E) and (F)) the ‘both’ prediction class is included in the results. Panels (A), (B) and (C) show the specificity when at 70%, 80% and 90% confidence levels, respectively. Panels (D), (E) and (F) show the specificity at 70%, 80% and 90% confidence levels, respectively.


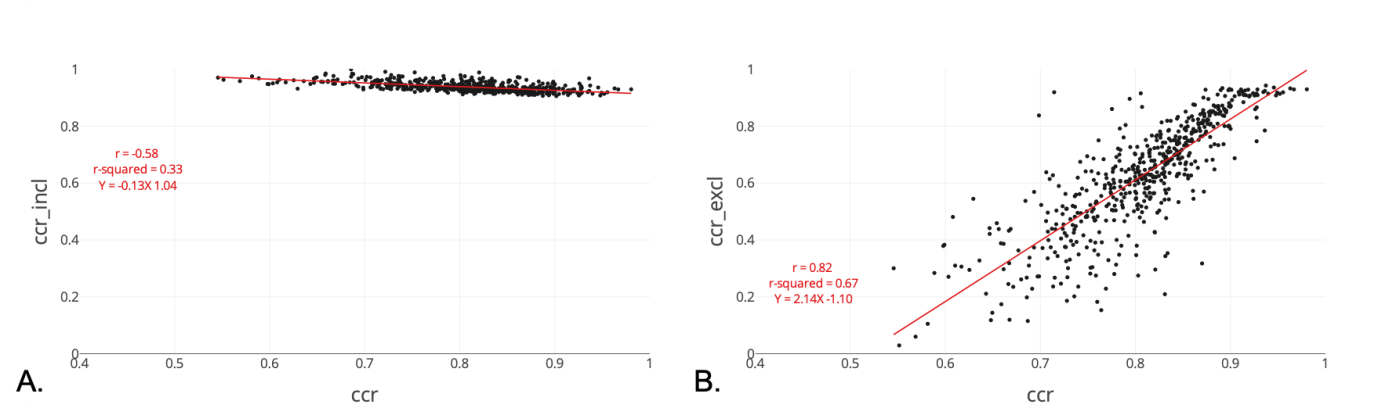


Figure S8: CCR comparison between results of QSAR and CP (confidence level 90%) models depending on whether (A) or not (B) the predictions assigned in the ‘both’ class are taken into account.


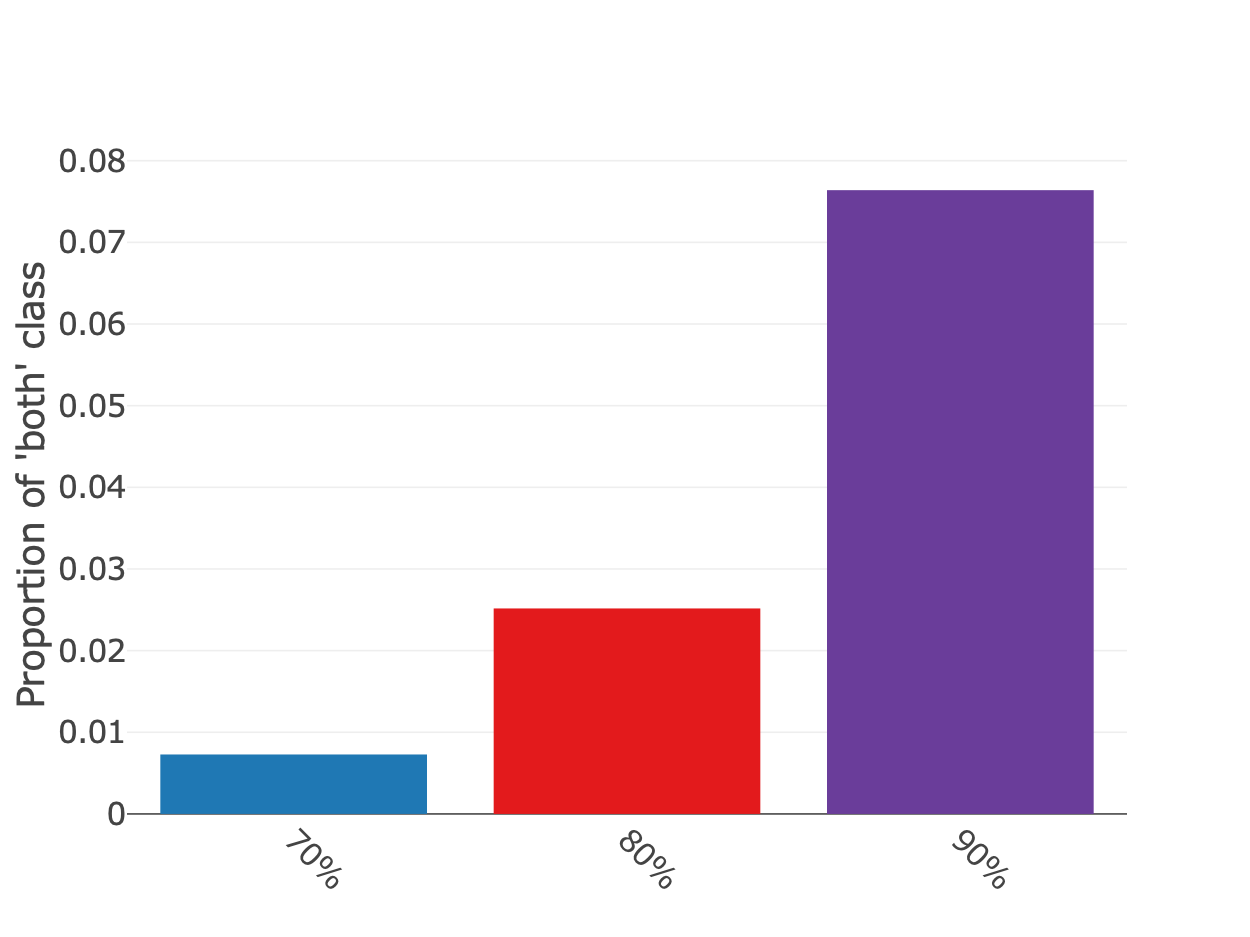


Figure S9: Proportion of the ‘both’ prediction category at different confidence levels for the temporal validation.


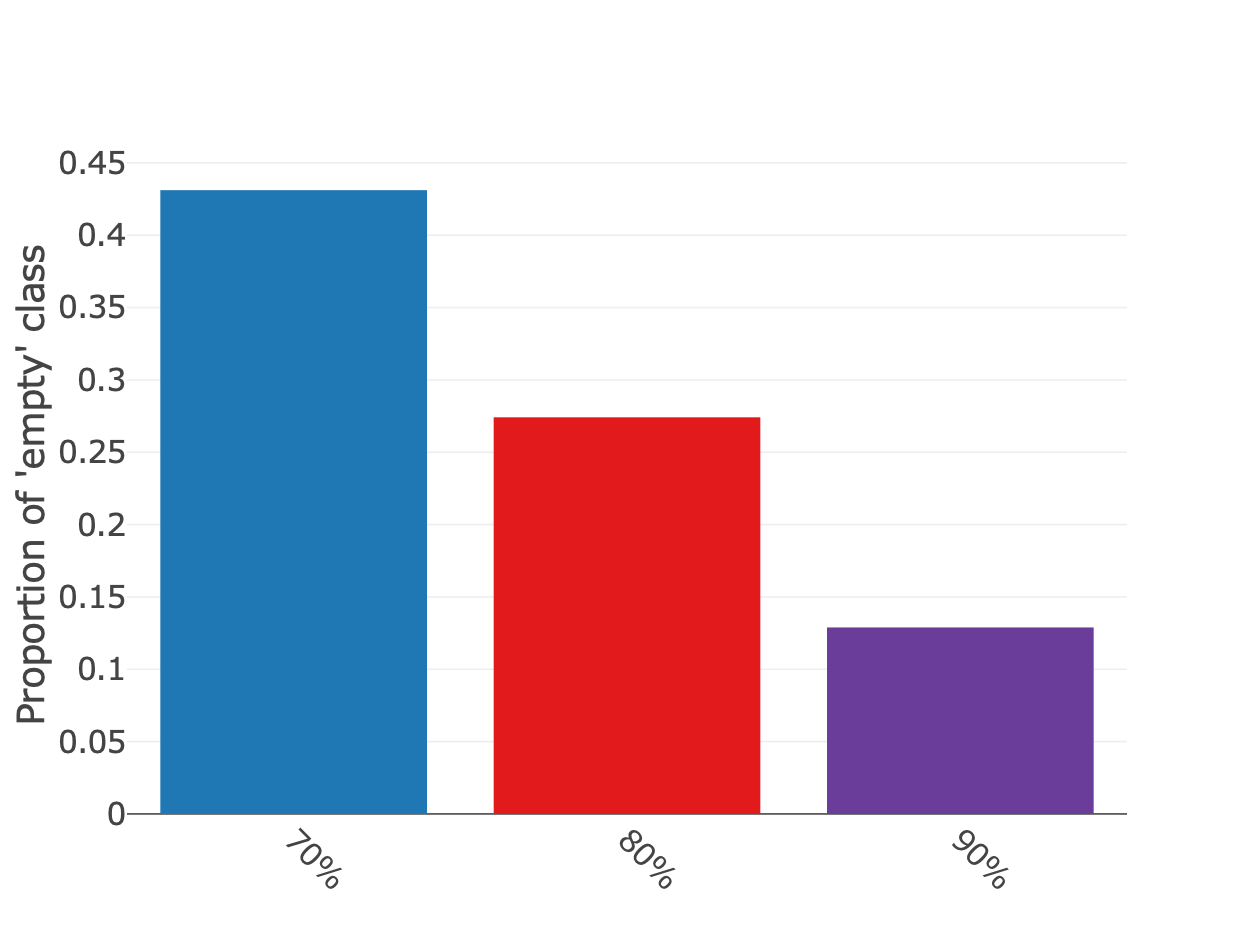


Figure S10: Proportion of the ‘empty’ prediction category at different confidence levels for the temporal validation.

Table S1: Selected target summary after the filtering steps.

| **Protein families** | **Initial selection** | **2 documents** | **Activity threshold** | **40 actives and 30 inactives** | **Final selection after the second selection round** | **Fraction of proteins**  **not selected** |
| --- | --- | --- | --- | --- | --- | --- |
| Enzyme > Kinase > Protein Kinase | 519 | 79 | 7.5 | 77 | 117 | 0.77 |
| Membrane receptor > Family A G protein-coupled receptor | 494 | 186 | 7 | 176 | 195 | 0.61 |
| Membrane receptor > Family B G protein-coupled receptor | 24 | 5 | 7 | 4 | 4 | 0.83 |
| Membrane receptor > Family C G protein-coupled receptor | 25 | 8 | 7 | 8 | 9 | 0.64 |
| Transcription factor > Nuclear receptor | 91 | 21 | 7 | 21 | 25 | 0.73 |
| Ion channel > Ligand-gated ion channel | 138 | 47 | 5 | 20 | 35 | 0.75 |
| Ion channel > Voltage-gated ion channel | 98 | 33 | 5 | 13 | 20 | 0.80 |
| Ion channel > Other ion channel | 11 | 5 | 5 | 5 | 5 | 0.55 |
| Others | 2643 | 449 | 6 | 351 | 378 | 0.86 |
|  | 4043 | 833 |  | 675 | 788 | 0.81 |

Protein targets, organised per protein families, selected after each filtering step. The selection directly from the ChEMBL database returns a total of 2643 unique protein targets. After filtering out those that are mentioned in less than 2 articles, this number drops to 833. There are 675 remaining protein targets after applying the protein family activity thresholds to consider only targets with at least 40 active and 30 inactive compounds. Eventually, performing a final selection on the targets rejected at the previous step using a standard activity threshold of 6.5 allows to select 788 unique protein targets (550 if only the human proteins are considered).

Table S2: Similarity comparison between the active compounds in the test set and the calibration for CHEMBL5451

| **Max similarity with actives in the calibration set** | **Molecule** | **p0** | **p1** |
| --- | --- | --- | --- |
| 0.16 | 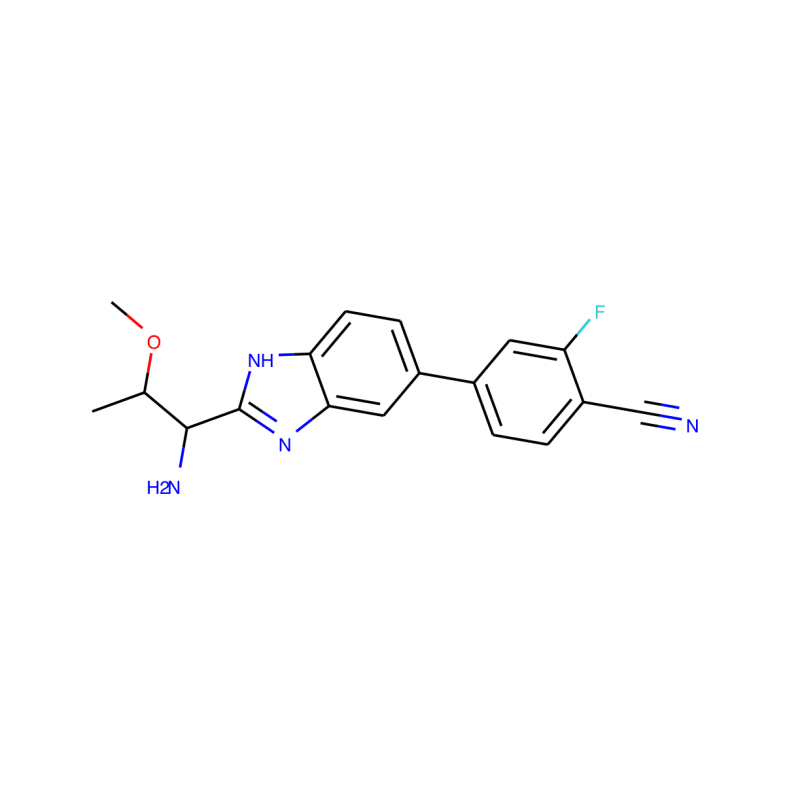 | 0.19 | 0.14 |
| 0.18 | 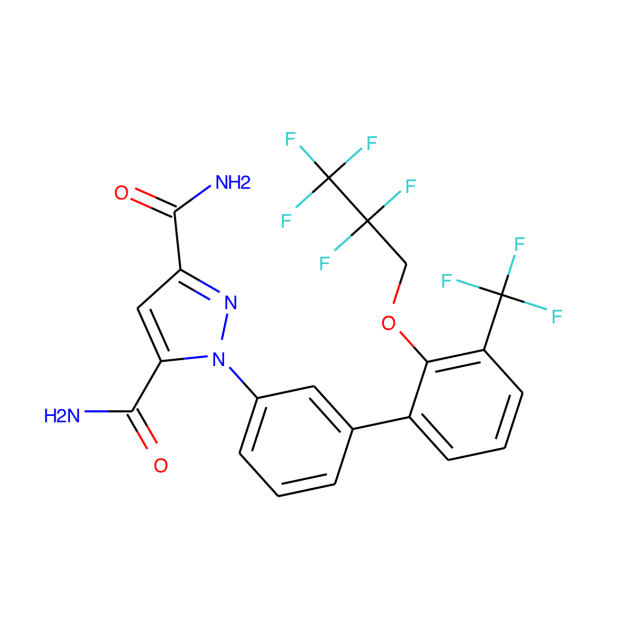 | 0.23 | 0.16 |
| 0.22 | 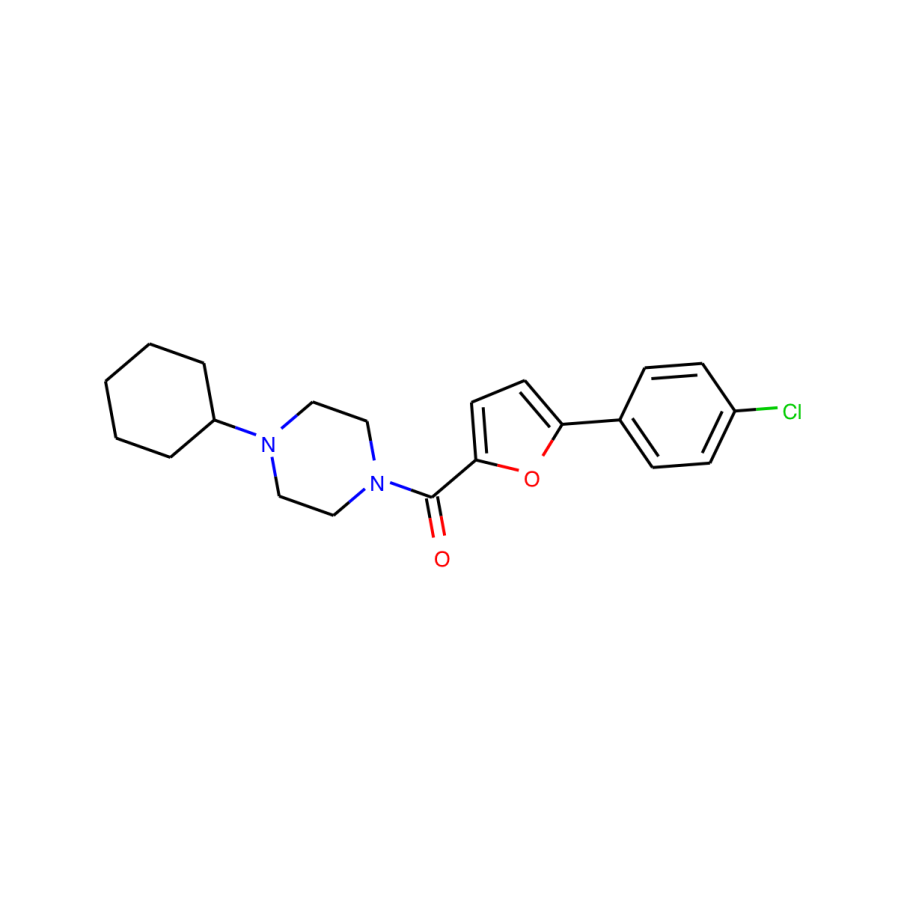 | 0.07 | 0.32 |
| 0.24 | 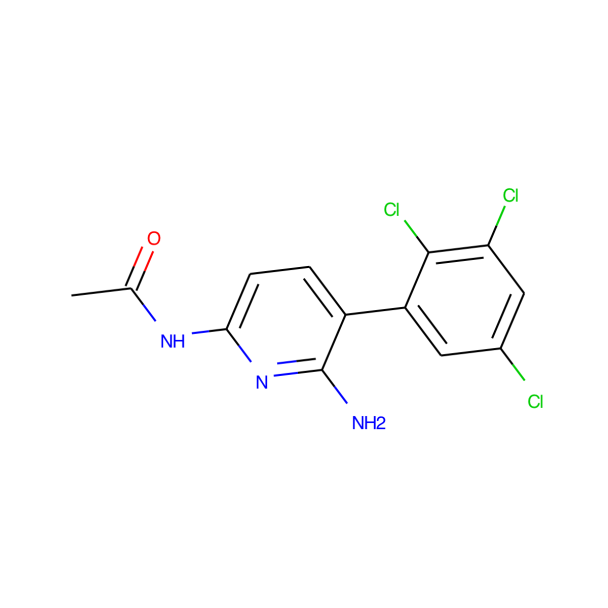 | 0.68 | 0.08 |
| 0.45 | 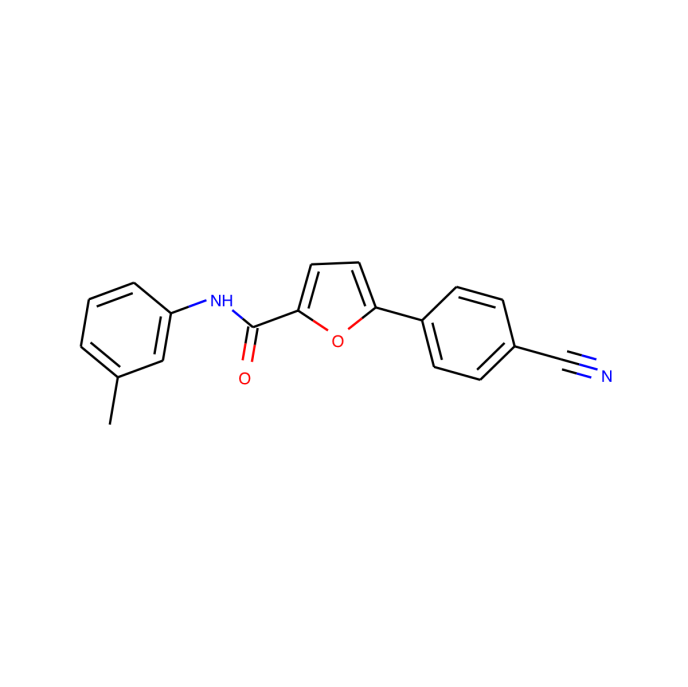 | 0.06 | 0.32 |
| 0.64 | 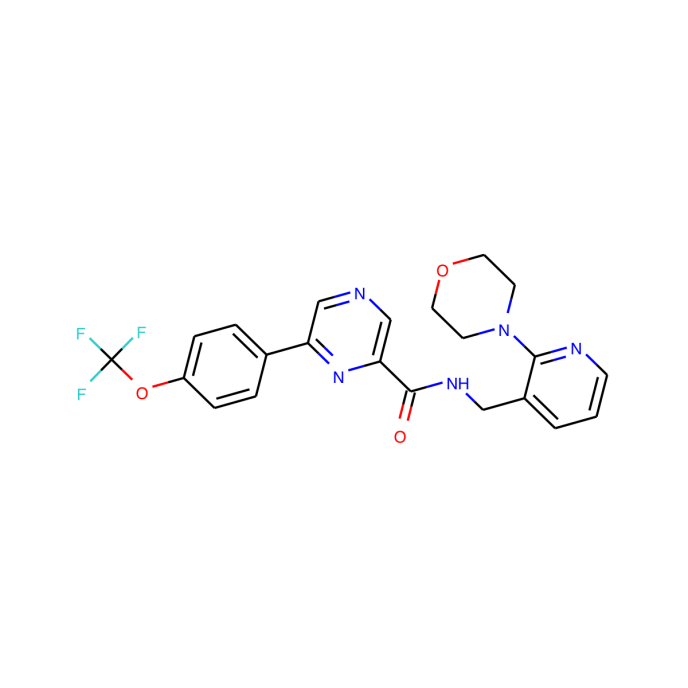 | 0.08 | 0.41 |
| 0.67 | 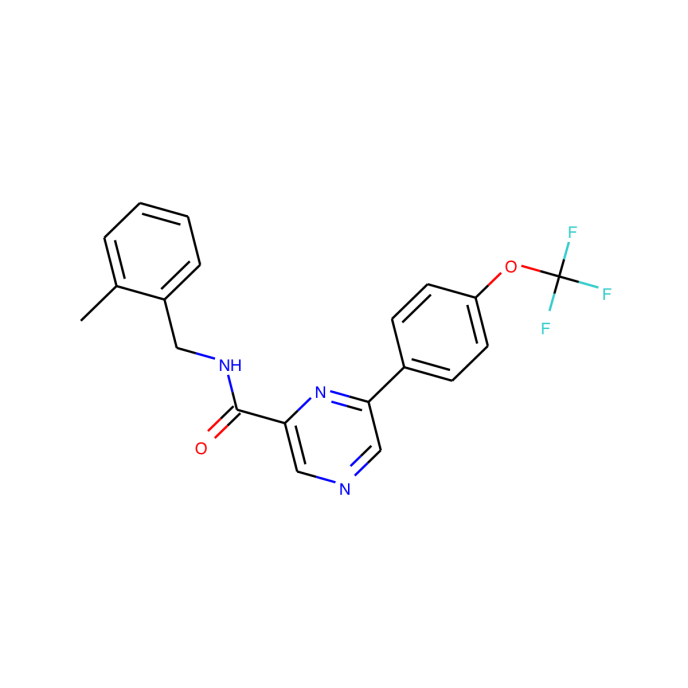 | 0.03 | 0.71 |
| 0.71 | 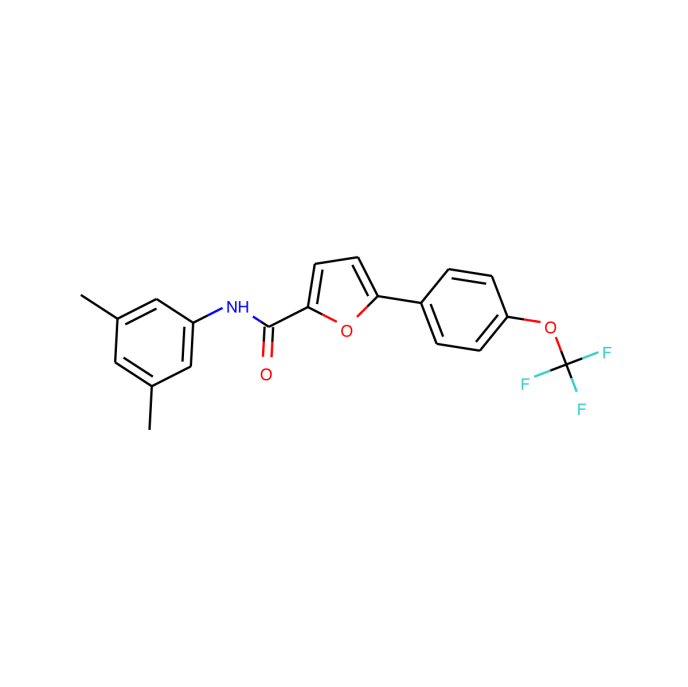 | 0.04 | 0.32 |
| 0.74 | 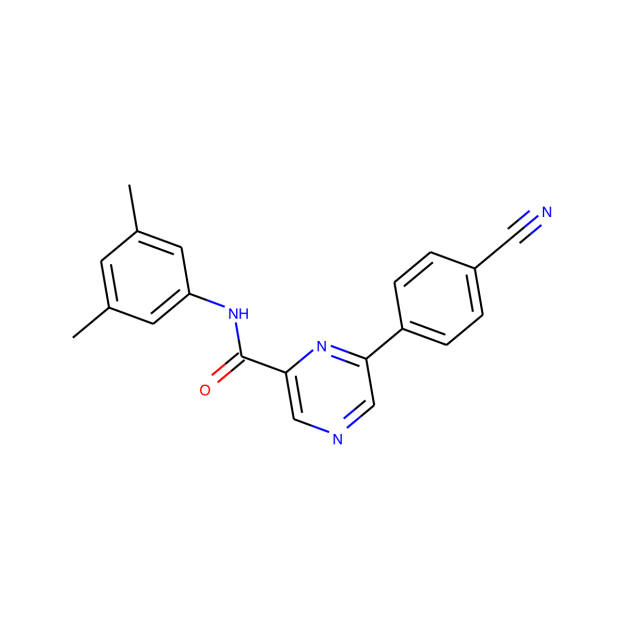 | 0.03 | 0.68 |
| 0.75 | 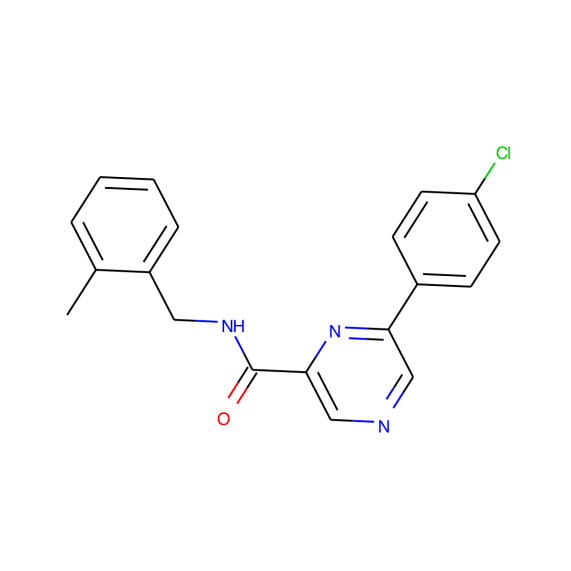 | 0.04 | 0.75 |
| 0.82 | 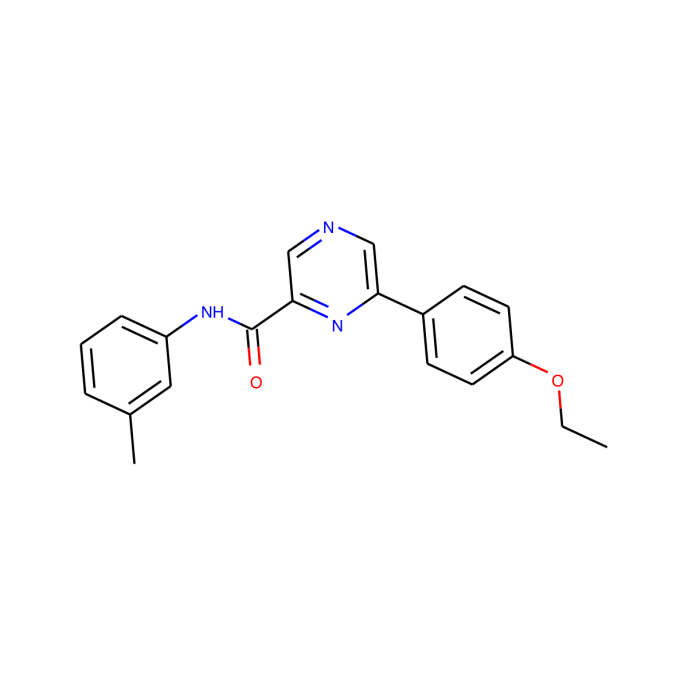 | 0.04 | 0.76 |

For each active compound represented, the Tanimoto coefficient for the most similar active compound in the calibration set is indicated in addition to the p-values returned by the conformal predictor for each class (p0: inactive, p1: active).

Table S3: Similarity comparison between the inactive compounds in the test set and the calibration for CHEMBL5451

| **Max similarity with inactives in the calibration set** | **Molecule** | **p0** | **p1** |
| --- | --- | --- | --- |
| 0.25 | 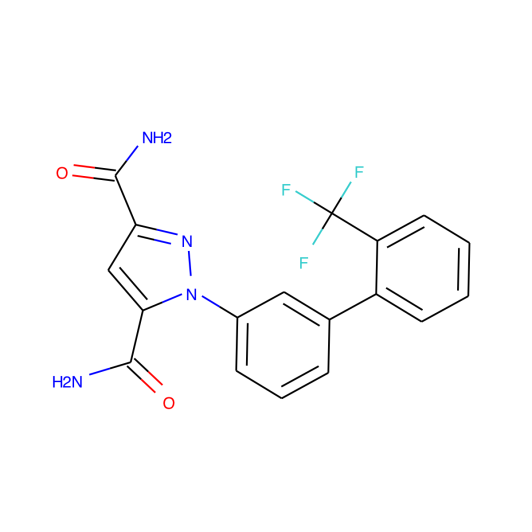 | 0.18 | 0.12 |
| 0.26 | 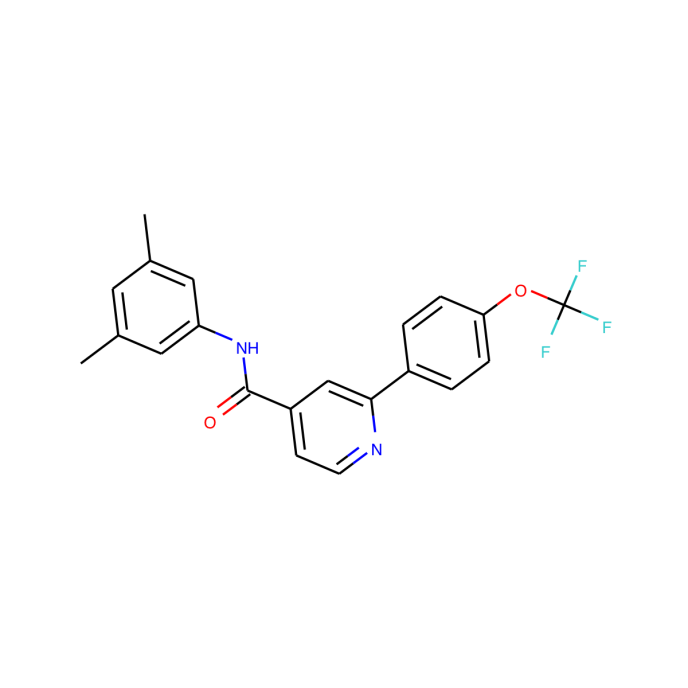 | 0.04 | 0.31 |
| 0.31 | 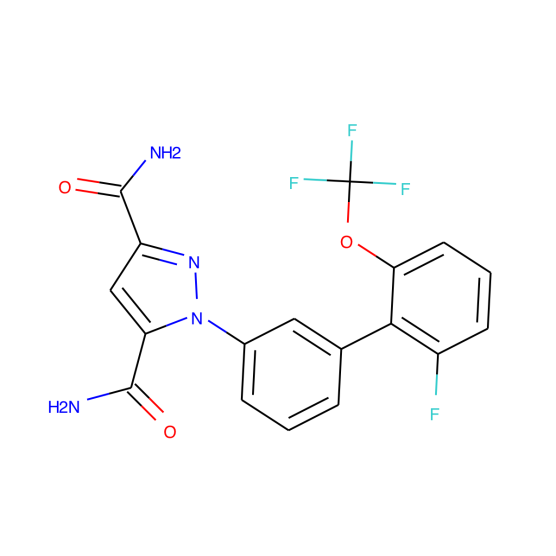 | 0.25 | 0.10 |
| 0.46 | 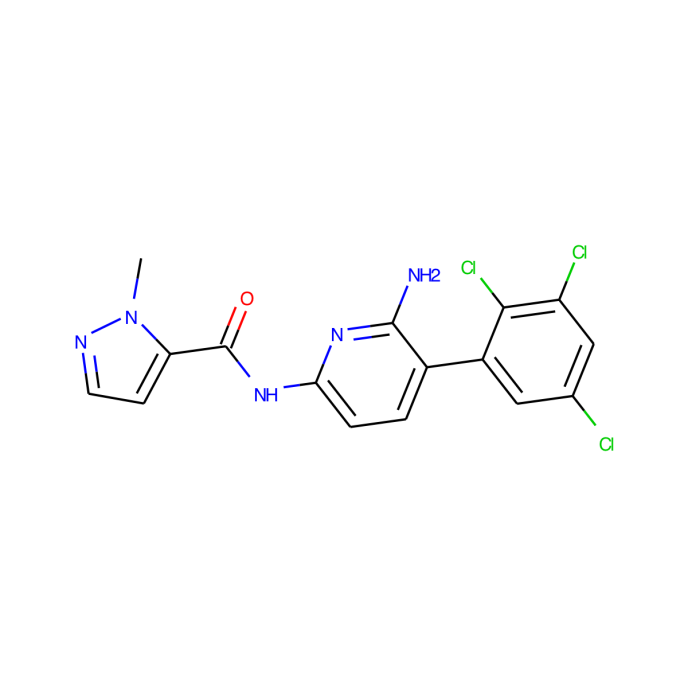 | 0.64 | 0.08 |
| 0.50 | 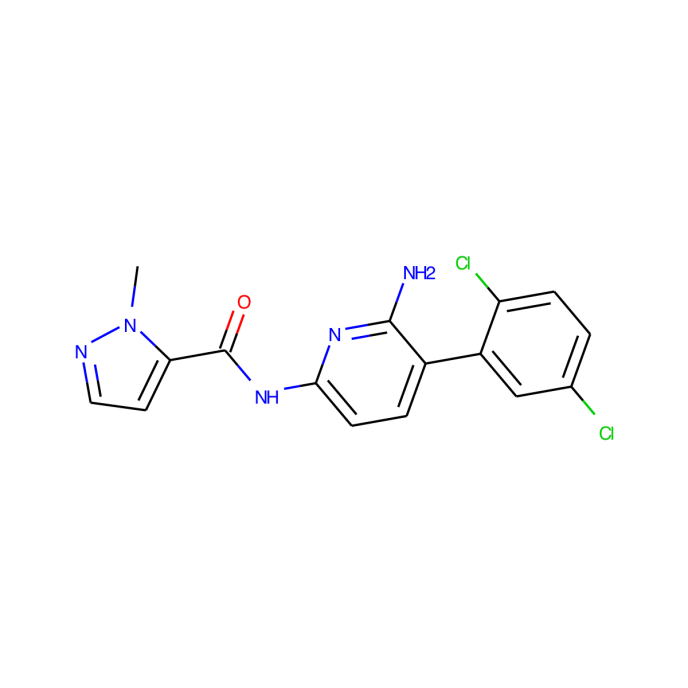 | 0.81 | 0.06 |
| 0.51 | 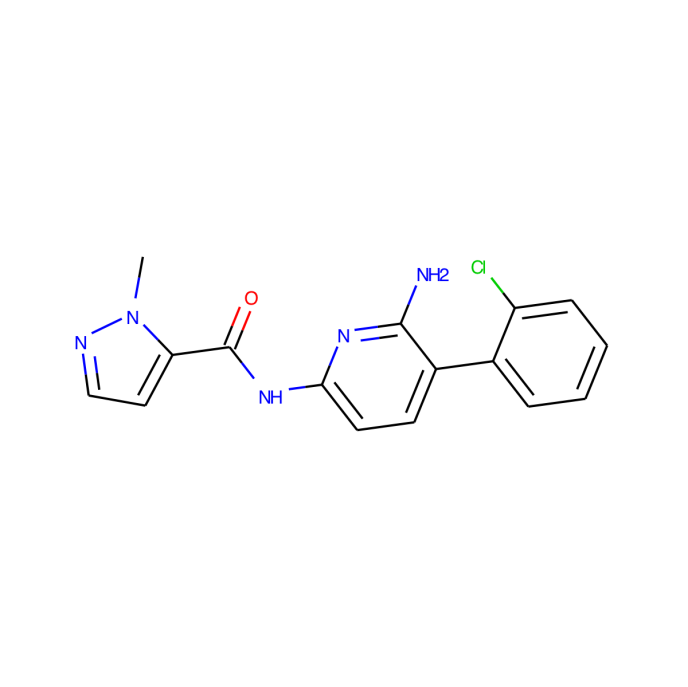 | 0.88 | 0.05 |
| 0.59 | 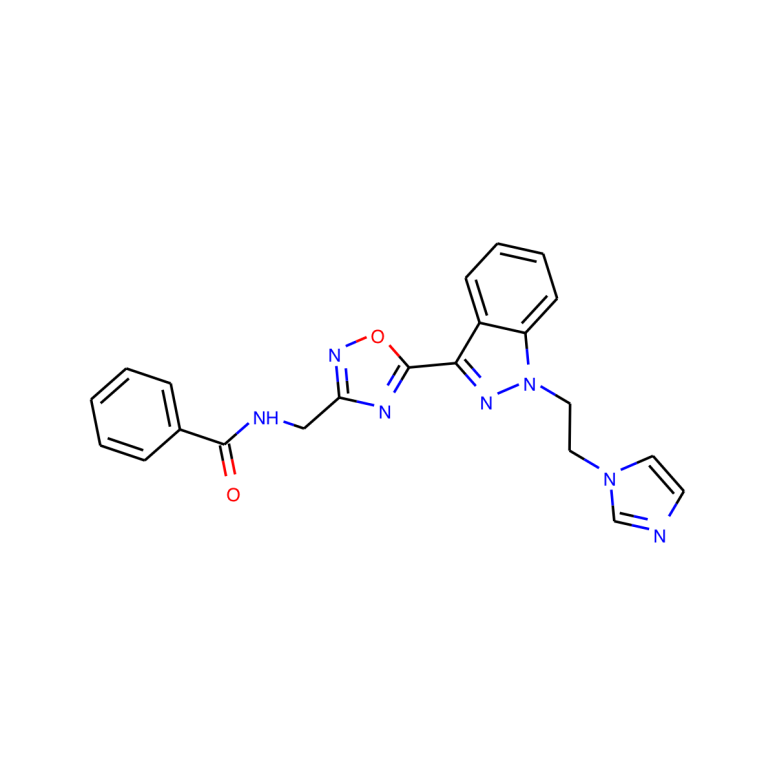 | 0.25 | 0.15 |
| 0.63 | 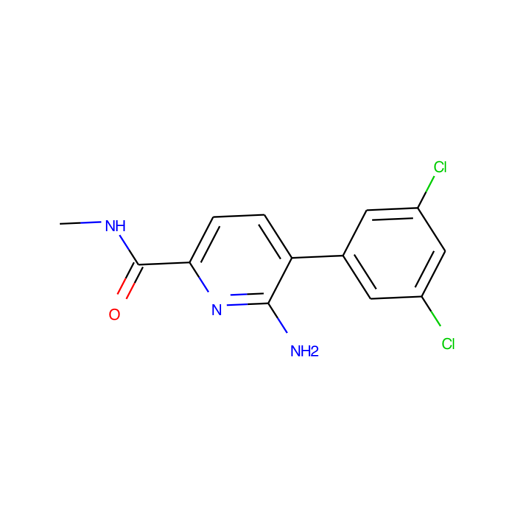 | 0.47 | 0.11 |
| 0.71 | 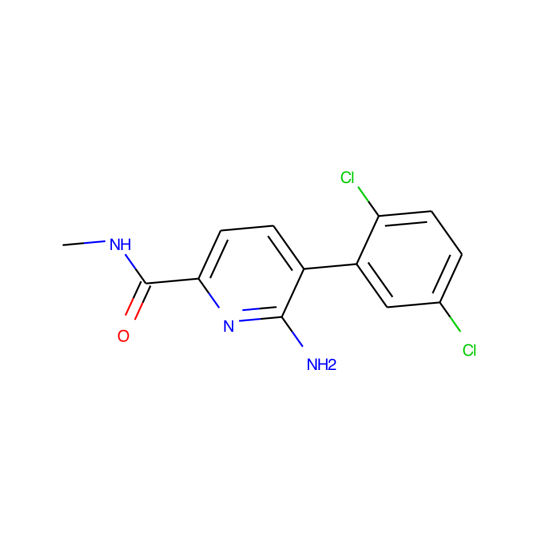 | 0.71 | 0.06 |
| 0.75 | 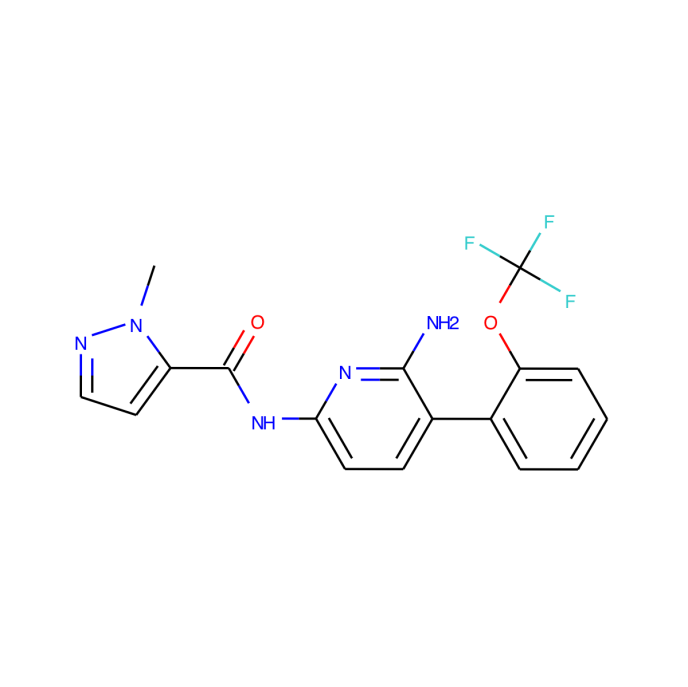 | 0.80 | 0.06 |

For each inactive compound represented, the Tanimoto coefficient for the most similar inactive compound in the calibration set is indicated in addition to the p-values returned by the conformal predictor for each class (p0: inactive, p1: active).

Table S4: Similarity comparison between the active compounds in the test set and the calibration for CHEMBL1955

| **Max similarity with actives in the calibration set** | **Molecule** | **p0** | **p1** |
| --- | --- | --- | --- |
| 0.14 | 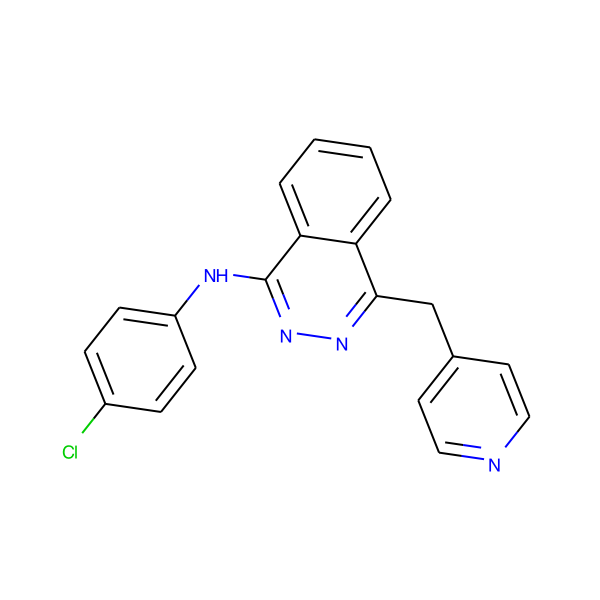 | 0.46 | 0.25 |
| 0.15 | 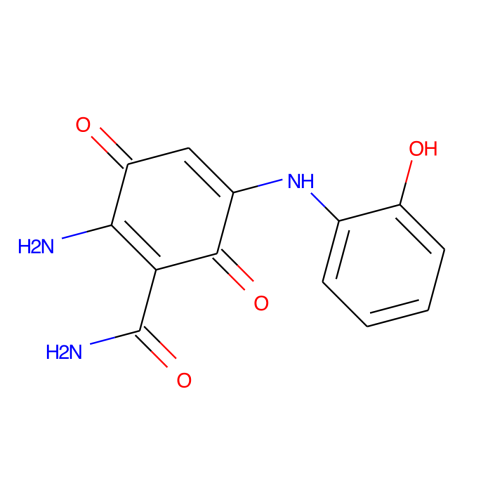 | 0.34 | 0.29 |
| 0.16 | 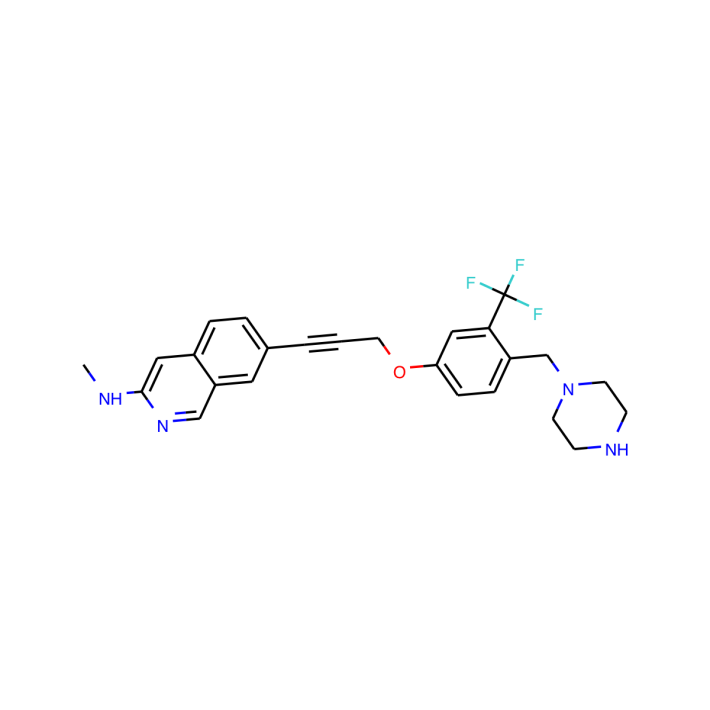 | 0.32 | 0.38 |
| 0.17 | 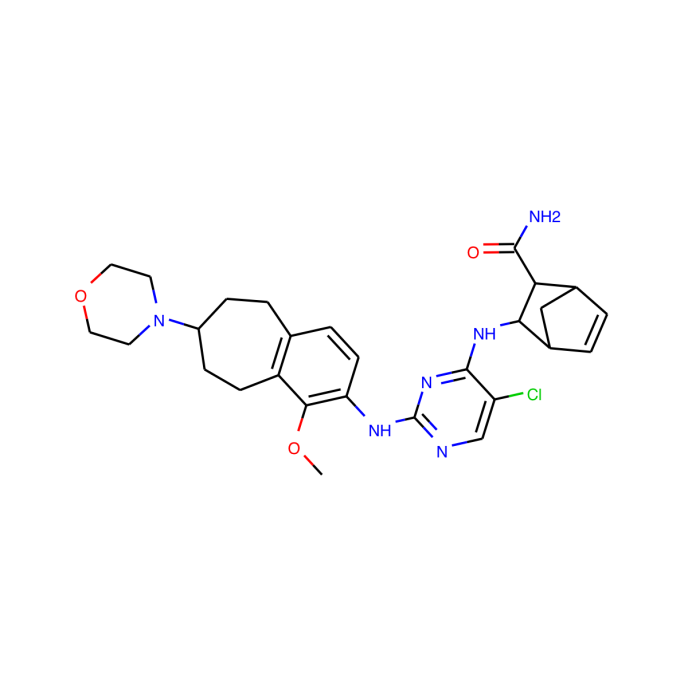 | 0.33 | 0.38 |
| 0.21 | 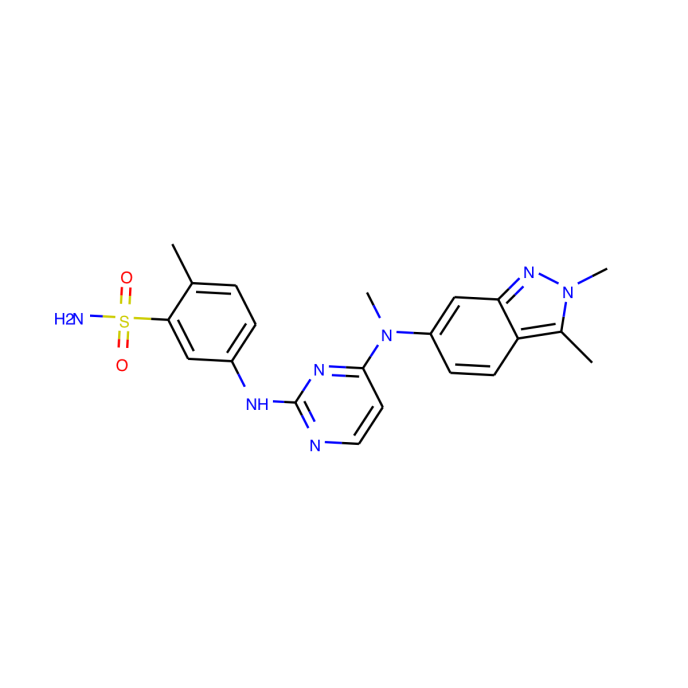 | 0.39 | 0.32 |
| 0.22 | 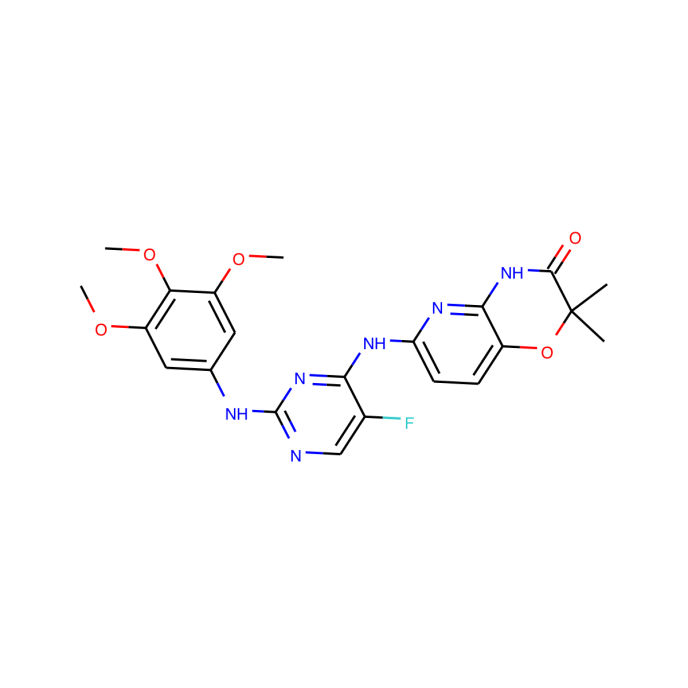 | 0.39 | 0.32 |
| 0.24 | 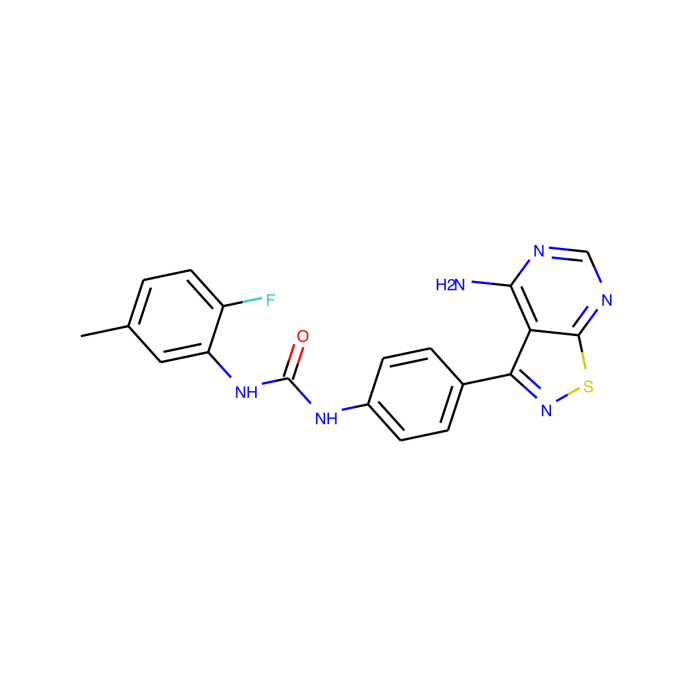 | 0.03 | 0.84 |
| 0.25 | 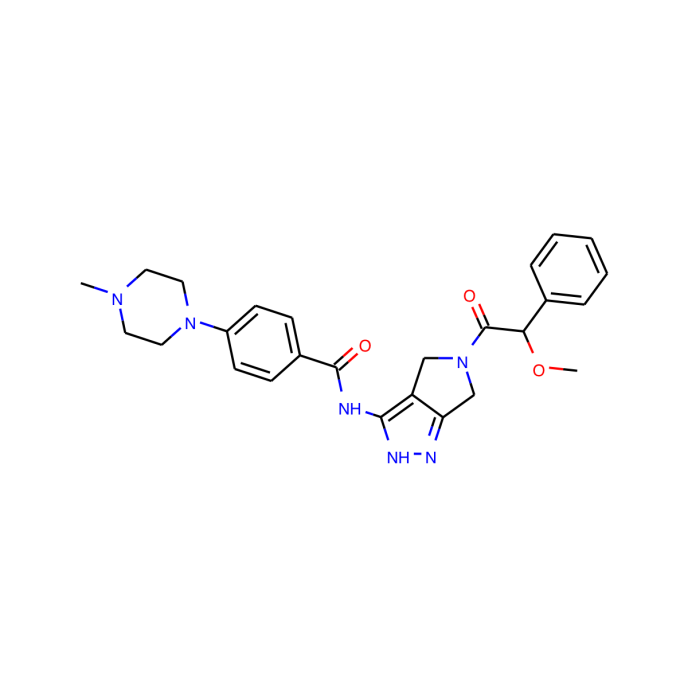 | 0.28 | 0.42 |
| 0.33 | 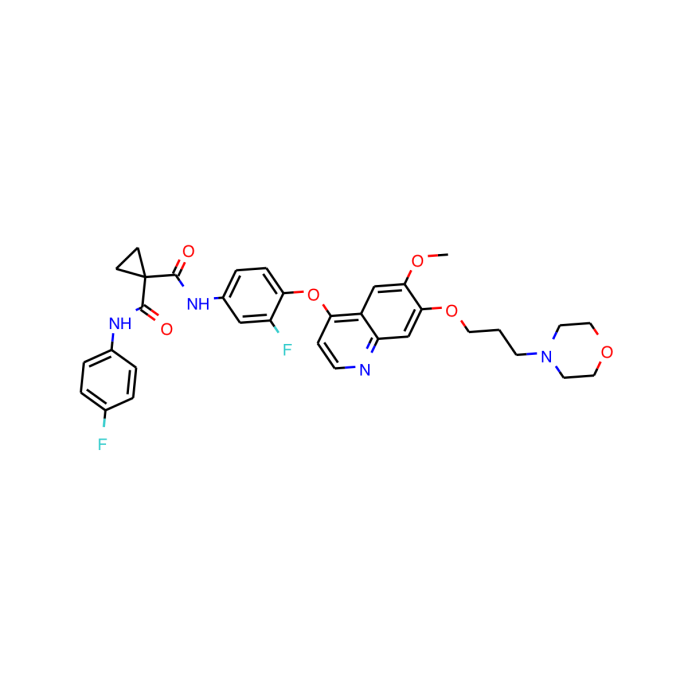 | 0.37 | 0.35 |
| 0.45 | 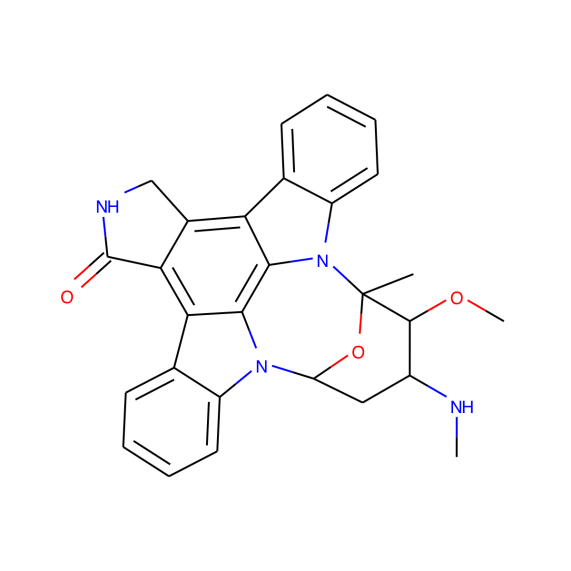 | 0.64 | 0.15 |
| 0.63 | 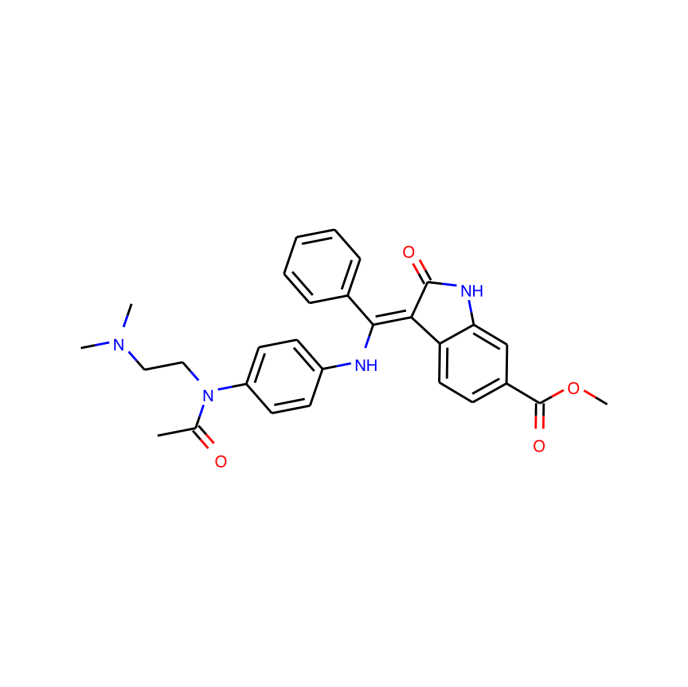 | 0.04 | 0.93 |
| 0.64 | 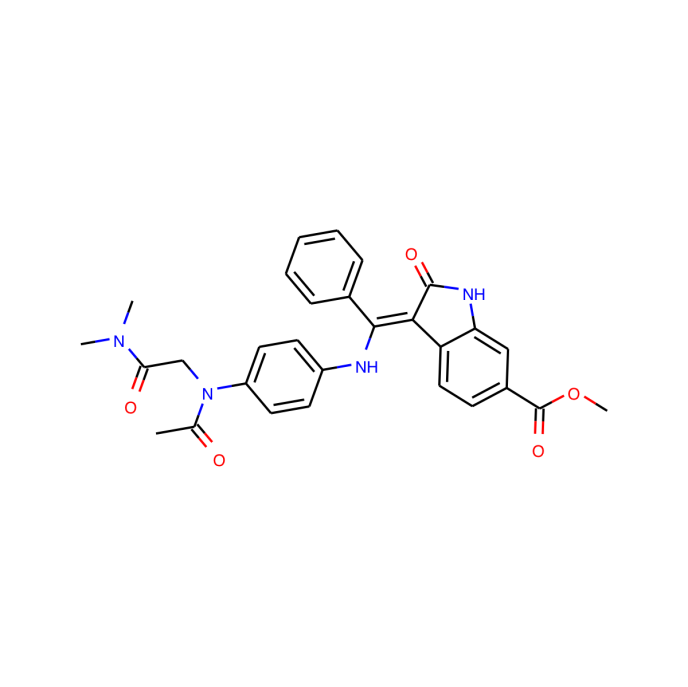 | 0.03 | 0.95 |
| 0.67 | 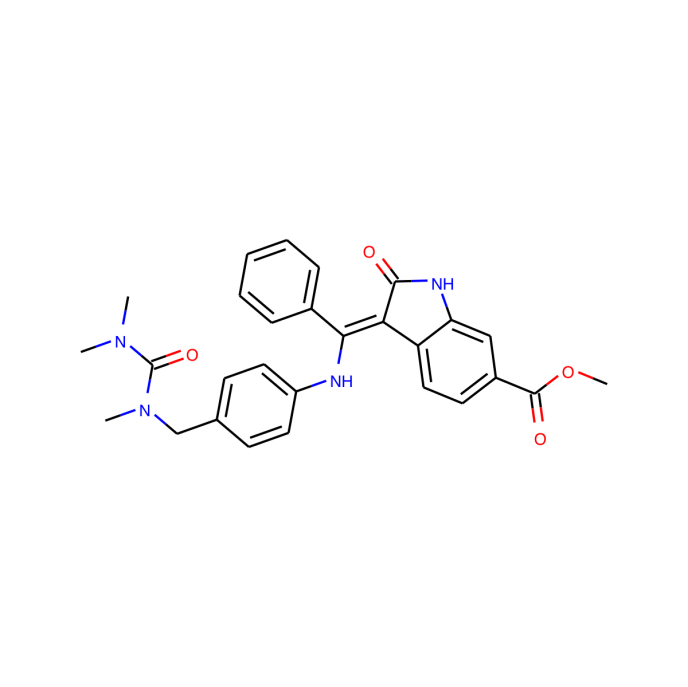 | 0.03 | 0.92 |

For each active compound represented, the Tanimoto coefficient for the most similar active compound in the calibration set is indicated in addition to the p-values returned by the conformal predictor for each class (p0: inactive, p1: active).

Table S5: Similarity comparison between the inactive compounds in the test set and the calibration for CHEMBL1955

| **Max similarity with actives in the calibration set** | **Molecule** | **p0** | **p1** |
| --- | --- | --- | --- |
| 0.11 | 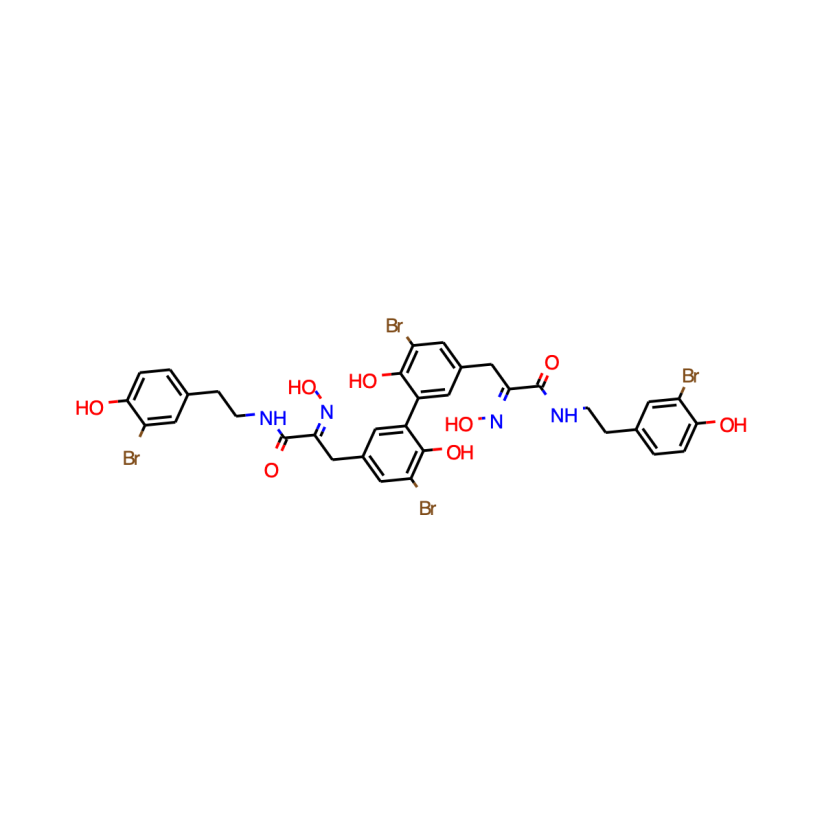 | 0.32 | 0.37 |
| 0.17 | 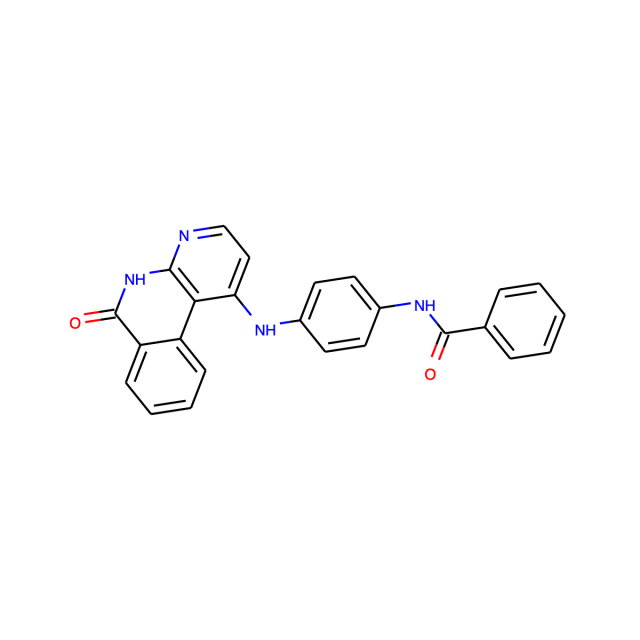 | 0.42 | 0.32 |
| 0.19 | 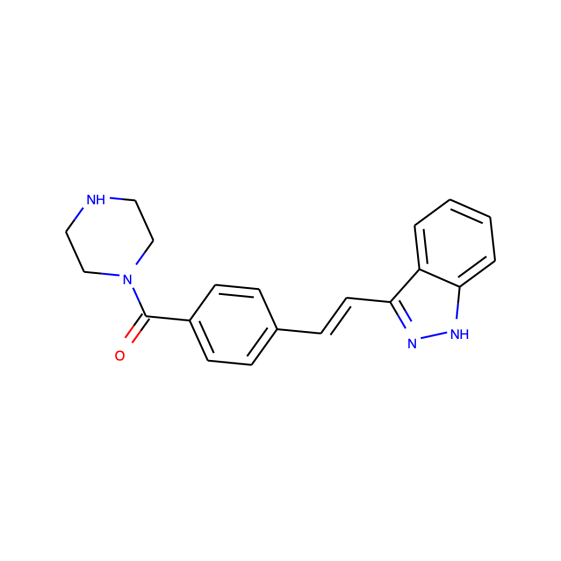 | 0.34 | 0.33 |
| 0.19 | 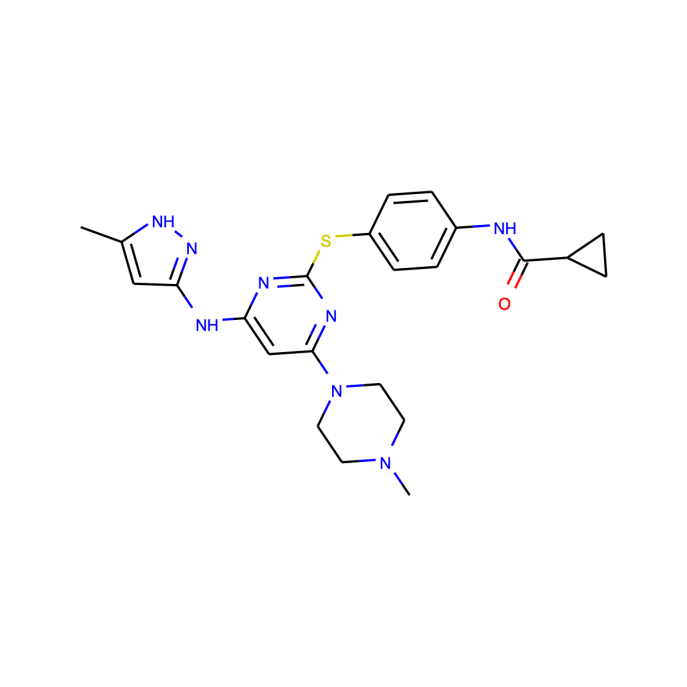 | 0.09 | 0.58 |
| 0.22 | 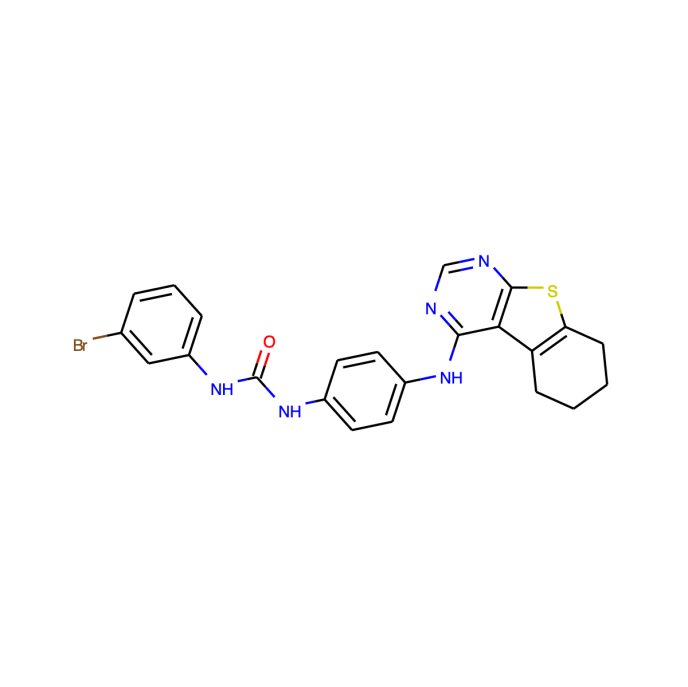 | 0.61 | 0.22 |
| 0.22 | 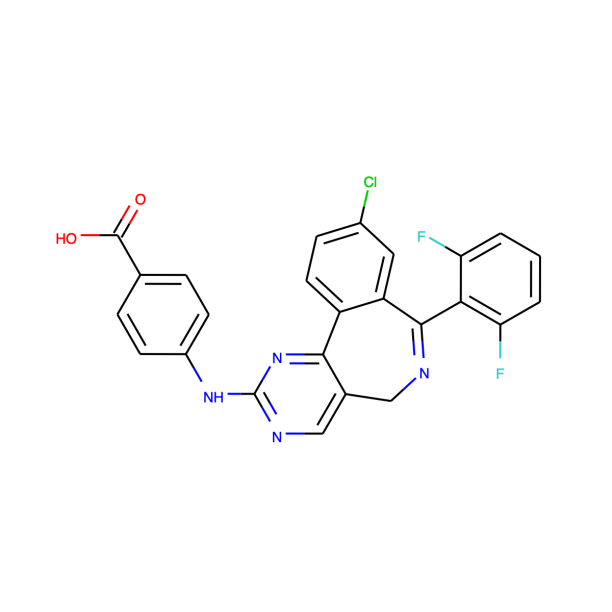 | 0.25 | 0.45 |
| 0.23 | 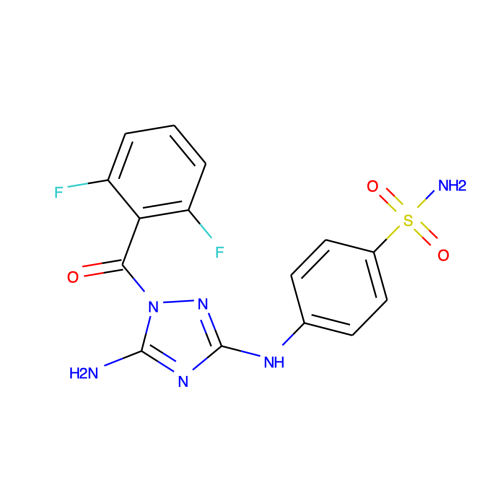 | 0.16 | 0.52 |
| 0.28 | 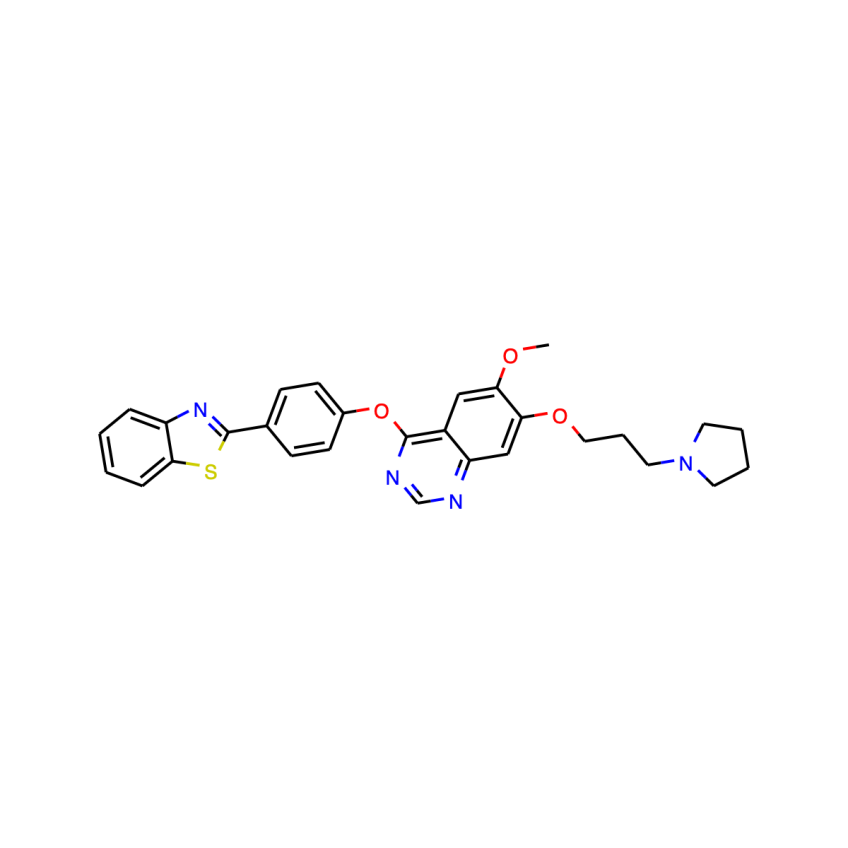 | 0.42 | 0.29 |
| 0.33 | 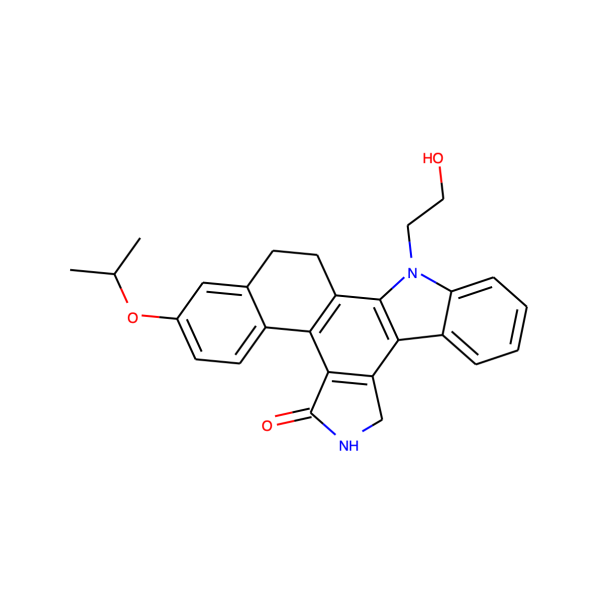 | 0.43 | 0.28 |
| 0.34 | 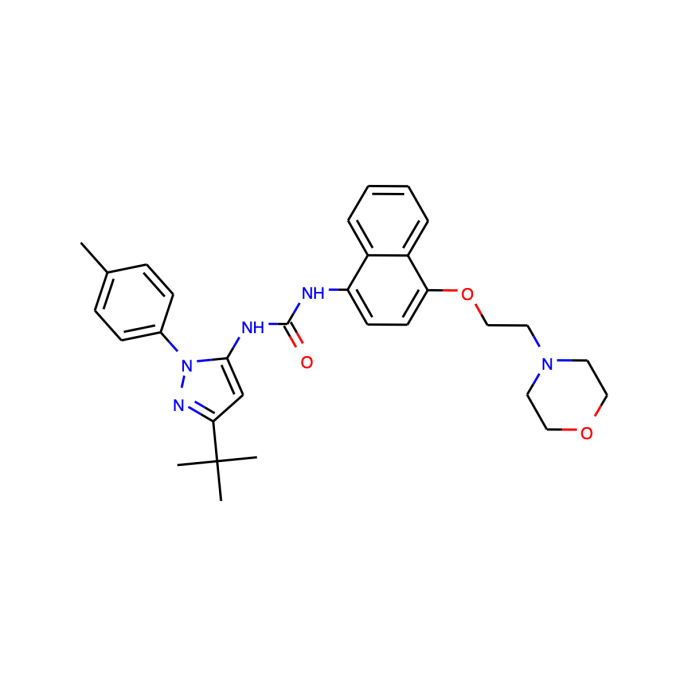 | 0.33 | 0.43 |
| 0.38 | 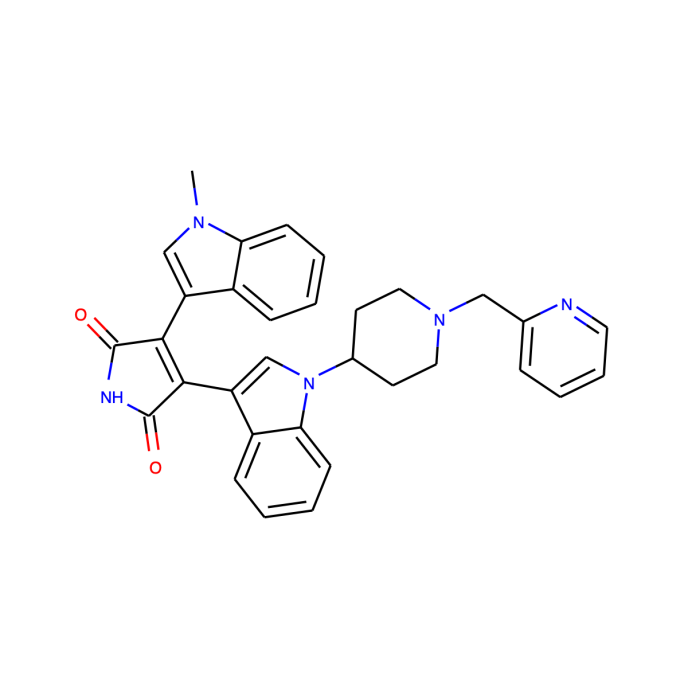 | 0.59 | 0.16 |
| 0.77 | 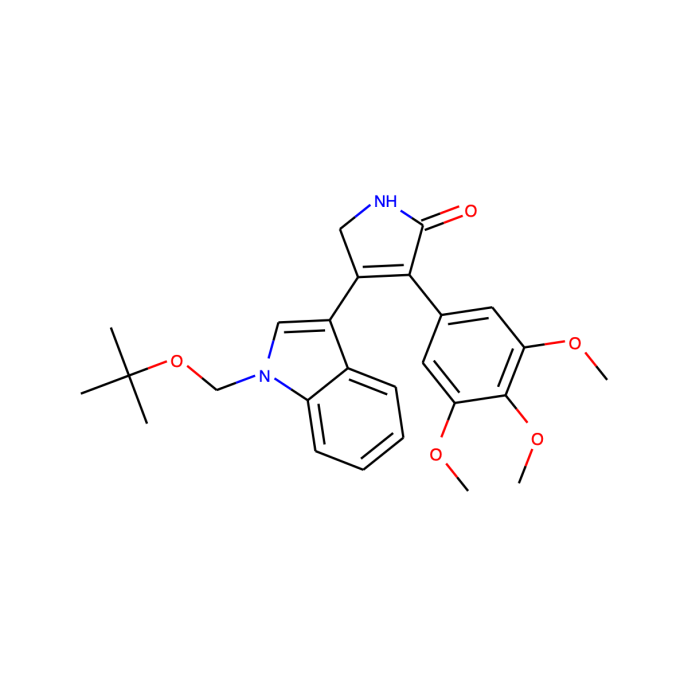 | 0.95 | 0.03 |

For each inactive compound represented, the Tanimoto coefficient for the most similar inactive compound in the calibration set is indicated in addition to the p-values returned by the conformal predictor for each class (p0: inactive, p1: active).
